# DNA Assembly in 3D Printed Fluidics

**DOI:** 10.1371/journal.pone.0143636

**Published:** 2015-12-30

**Authors:** William G. Patrick, Alec A. K. Nielsen, Steven J. Keating, Taylor J. Levy, Che-Wei Wang, Jaime J. Rivera, Octavio Mondragón-Palomino, Peter A. Carr, Christopher A. Voigt, Neri Oxman, David S. Kong

**Affiliations:** 1 MIT Media Lab, School of Architecture and Planning, Massachusetts Institute of Technology, Cambridge, MA, United States of America; 2 Department of Biological Engineering, Massachusetts Institute of Technology, Cambridge, MA, United States of America; 3 Department of Mechanical Engineering, Massachusetts Institute of Technology, Cambridge, Massachusetts, United States of America; 4 Massachusetts Institute of Technology Lincoln Laboratory, Lexington, MA, United States of America; Northeastern University, UNITED STATES

## Abstract

The process of connecting genetic parts—DNA assembly—is a foundational technology for synthetic biology. Microfluidics present an attractive solution for minimizing use of costly reagents, enabling multiplexed reactions, and automating protocols by integrating multiple protocol steps. However, microfluidics fabrication and operation can be expensive and requires expertise, limiting access to the technology. With advances in commodity digital fabrication tools, it is now possible to directly print fluidic devices and supporting hardware. 3D printed micro- and millifluidic devices are inexpensive, easy to make and quick to produce. We demonstrate Golden Gate DNA assembly in 3D-printed fluidics with reaction volumes as small as 490 nL, channel widths as fine as 220 microns, and per unit part costs ranging from $0.61 to $5.71. A 3D-printed syringe pump with an accompanying programmable software interface was designed and fabricated to operate the devices. Quick turnaround and inexpensive materials allowed for rapid exploration of device parameters, demonstrating a manufacturing paradigm for designing and fabricating hardware for synthetic biology.

## Introduction

Synthetic Biology is a rapidly advancing field that is being used to create novel biotechnology applications, next-generation therapeutics, and new methods of scientific inquiry [1–3]. The commercialization and rapid decline in price of DNA sequencing and synthesis technologies have enabled much of this development. Solid-phase DNA synthesis has declined in price, enabling researchers to routinely design and order synthetic DNA up to several kilobases in length. However, assembly of these molecules into larger constructs remains an essential technique. Indeed, DNA assembly is necessary to generate complex single-gene and multi-gene constructs [4–6], to create functionally-diverse part combinations (e.g., gene clusters with libraries of RBSs and promoters [7,8]), to “shuffle” homologous proteins at specific recombination points (e.g., shuffling of three trypsinogen homologues [9]), and to explore higher-order effects of genetic architectures (e.g., the position and orientation of transcription units [7]). Recent advances in “one-pot” DNA assembly methods, such as Golden Gate assembly [10], have made assembling complex genetic constructs simpler.

Most practitioners perform DNA assembly reactions by traditional laboratory techniques employing manual pipettors. Microfluidics presents an opportunity to automate and parallelize DNA assembly reactions and reduce reagent volumes. Researchers have previously demonstrated synthesis of several distinct genes in parallel microfluidic reactors (as small as 500 nL)[11–13]. These devices employed polymerase construction and amplification (PCA) to assemble genes from pools of oligonucleotides, a process that requires *in situ* thermocycling. More recently, researchers have demonstrated one-pot and hierarchical DNA assembly in microfluidic devices to generate libraries of DNA constructs [14]. All devices used for gene synthesis and DNA assembly have been manufactured using polydimethylsiloxane (PDMS) replica molding, a common approach for microfluidic device fabrication.

Design and fabrication of molded PDMS devices is time-consuming and expensive. A recent study estimates that the labor and material costs of a new single layer PDMS microfluidic is one day, and $215 [15]. The cost and complexity of fabricating and using PDMS microfluidic devices has slowed adoption even though the technology shows tremendous potential for the miniaturization and fine control of liquids. The duration, complexity and cost of the design, build, and test cycle for replica molded PDMS microfluidics makes rapid prototyping challenging and limits the accessibility of this technology for non-experts.

3D printing provides a potential one-step manufacturing approach for fabricating microfluidic devices to run DNA assembly reactions. Micro- and millifluidics have been printed using fused-deposition modeling (FDM) of thermoplastics [16,17], stereolithography (SLA) [15,18–21], and inkjet printing of photo-curable polymers [22–25]. Devices have been built for less than $1 per device in only a few hours [16],[19]. Numerous desktop FDM and SLA printers are marketed between $349 to $6000 [26–28]. On-demand 3D printing services, such as Shapeways, can print objects in a range of plastic polymers at a material cost between $0.28–5.99 per cubic centimeter [29]. Internal channel diameters in 3D printed microfluidics have been reported as low as 100 microns [24]. However, DNA assembly has not been demonstrated in 3D printed fluidics. Moreover, the compatibility of 3D-printed materials with nucleic acids has not been reported.

3D printing has another additional advantage for printing fluidics. 3D design files are easily shared on the web, allowing practitioners with access to a 3D printer to download and print fluidic devices and collaborate on new designs. There are many examples of 3D-printed labware that are shared on the web, including a centrifuge (DremelFuge [30]), optics equipment [31], syringe pumps [32]-[33], a colorimeter [34], and a turbidostat [35]. Our goal here is to allow synthetic biologists to download and 3D print devices that can be used to parallelize and automate DNA assembly reactions.

**Fig 1:**
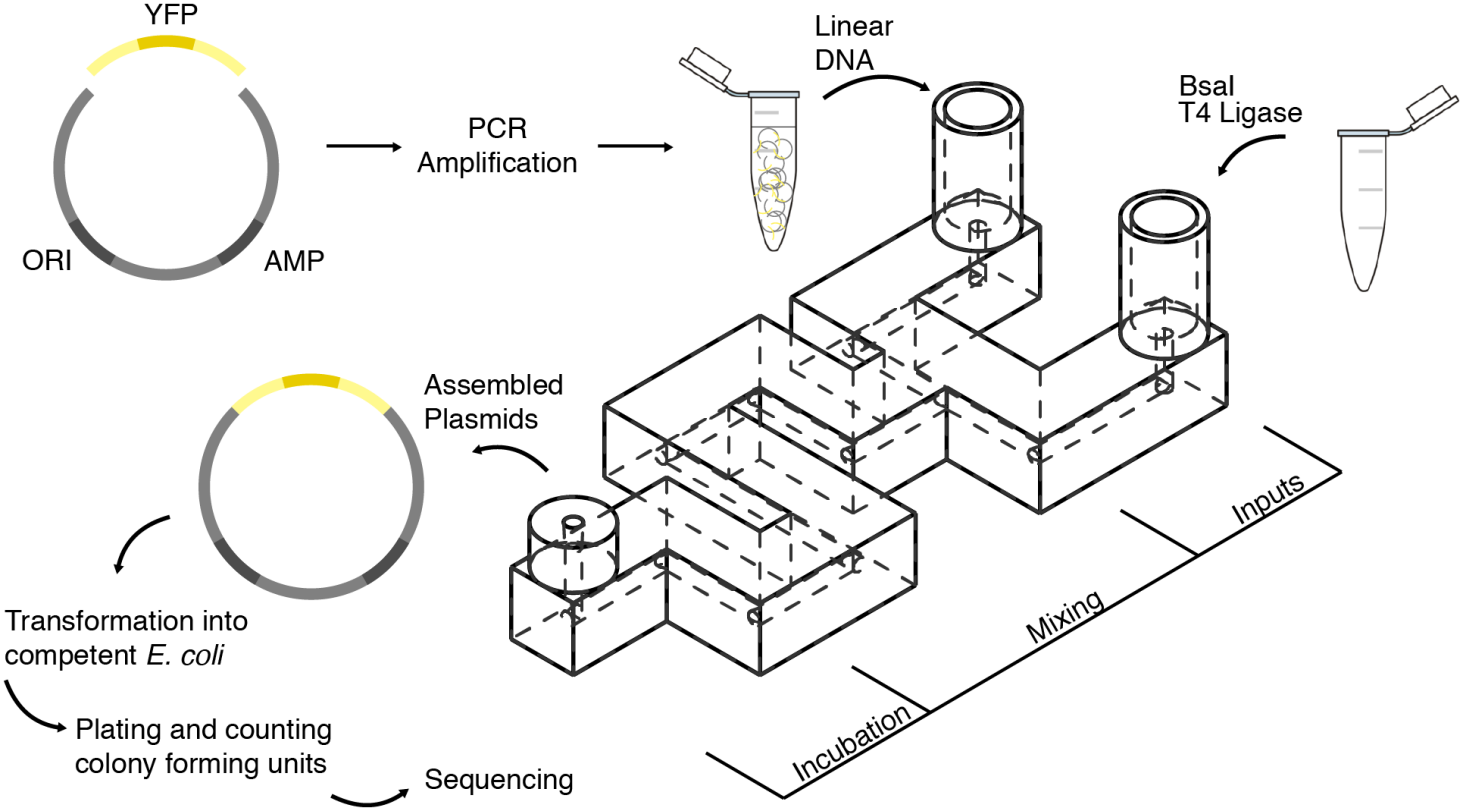
DNA assembly in 3D-Printed Fluidics. Two linear DNA segments were used, a constitutively expressed yellow fluorescent protein (YFP) reporter, and a plasmid backbone containing an ampicillin selection marker (AMP) and origin of replication (ORI). Each linear segment was amplified using polymerase chain reaction (PCR), prior to assembly. The linear segments were inserted into one inlet of the fluidic device. Golden Gate reagent mix (enzymes BsaI and T4 Ligase, in T4 Ligase buffer) were inserted into the other. The two inputs were pulled into the device and mixed via co-laminar diffusion. After a 90-minute room temperature incubation, the reaction products were removed from the device and then transformed into *E*. *coli*. This experiment was performed with all three device designs (Fig 2). To control for device mixing efficacy, assemblies were also performed in the devices in which the linear DNA segments and Golden Gate enzyme mix were mixed off-device prior to the experiment. Detailed visual protocols for running this experiment in the SW-FUD devices can be found in S1 Fig.

In this work, we demonstrate Golden Gate DNA assembly in 3D-printed fluidic devices (Fig 1). We printed the devices using two methods: a desktop SLA 3D printer (Form 1+, Form Labs, Somerville, Massachusetts) and an on-demand 3D printing service (Shapeways, New York, New York). The design files for all devices can be found in the website ‘Metafluidics’ (www.metafluidics.com). The smallest printed device had a channel width of 220 microns and reactor volume of 490 nL. We designed the micro- and millifluidics to mix linear double stranded DNA with enzymes to assemble plasmids via Golden Gate biochemistry. The assembly reactions occurred at room temperature, eliminating the need for an incubator. We then transformed the assembled plasmids into *E*. *coli* and compared the number of transformants to a tube control.

A bespoke, 3D-printed syringe pump operated the fluidic devices (S5–S10 Figs). The pump’s mechanical components were 3D printed using a desktop FDM 3D printer (MakerBot Replicator 2, MakerBot Industries, Brooklyn, New York) and the pump was controlled by a custom control board (S9 Fig) fabricated using a desktop CNC mill (Roland Modela MDX-20 CNC mill, Roland DG Corporation, Hamamatsu, Japan) running Arduino firmware. A user interface (S10 Fig) enabled the user to control the pump via USB (S6 Fig).

We designed both the fluidic devices and syringe pump using rapid and collaborative design iterations, enabled by the use of desktop 3D printers and on-demand printing services. We tested and re-designed each fluidic device multiple times before arriving at the final forms (S4 Fig). The final designs of the 3D-printed micro- and millifluidic mixers, the syringe pump, and the control software are available in the supplementary information.

## Results

### 3D printed micro- and millifluidic device design and characterization

**Fig 2:**
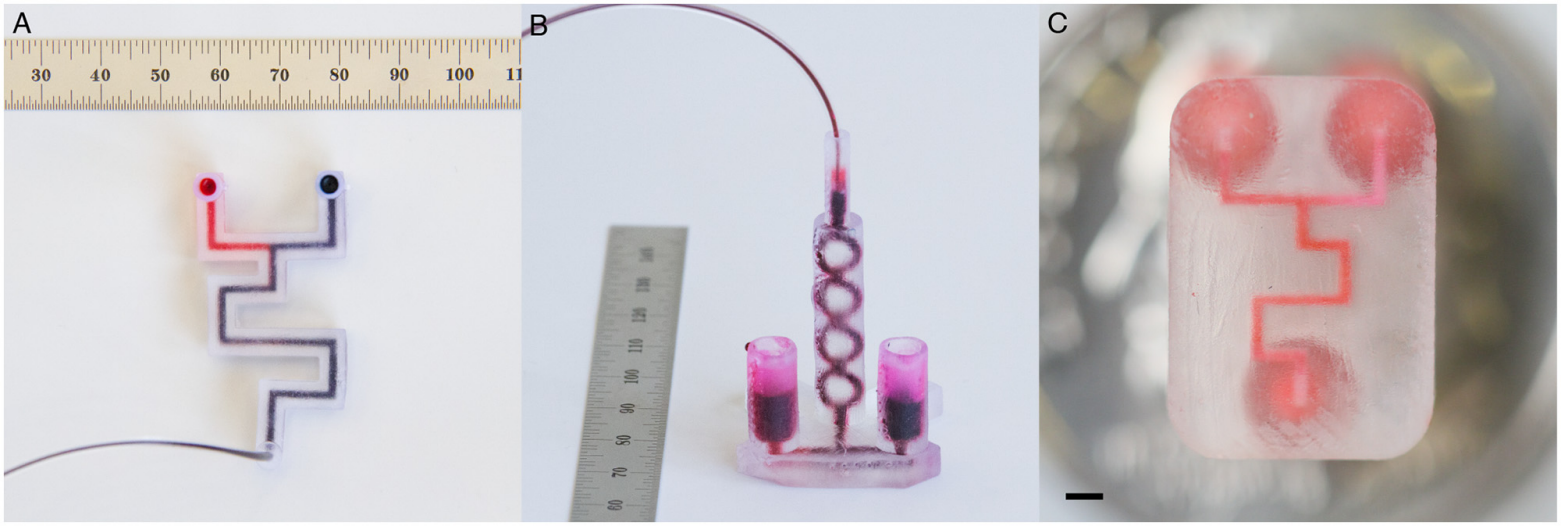
Fluidic devices printed using the Form 1+ and Shapeways Frosted Ultra Detail. DNA assembly was tested in three fluidic device designs: (A) the Form 1+ Co-Laminar Mixer, (B) the Form 1+ 3D Micromixer, and (C) the SW-FUD Co-Laminar Mixer (scale bar: 1 mm). The Form 1+ devices were designed with 1.5 mm diameter circular channels and the SW-FUD device was designed with a 300-micron square channel.

Three fluidic devices were designed and fabricated to conduct DNA assemblies: a Co-Laminar Mixer printed on the Form 1+, a 3D Micromixer printed on the Form 1+, and a Co-Laminar Mixer printed using Shapeways Frosted Ultra Detail material (SW-FUD) (Fig 2). Each printing method has advantages. The Form 1+ affords rapid and inexpensive design iterations and near optical clarity using the “Clear” Photoactive Resin. The build volume of the Form1+ is 125 x 125 x 165 mm (X x Y x Z) and the manufacturer claims a minimum lateral laser beam size of 10 microns and feature size of 300 microns [27]. The PreForm software package for the Form1+ offers four z-resolution steps for printing: 25, 50, 100 and 200 microns. We found no measurable difference in resolution between 25 and 50 microns; a 50-micron step size was used for all prints. For printing internal channels, no internal supports were required.

The highest resolution printing material offered by Shapeways is Frosted Ultra Detail (FUD), a UV-cured acrylic polymer. The minimum embossed or engraved feature for SW-FUD is 100 microns. The printer accuracy is +/- 25 to 50 microns for every 2540 microns [29]. Frosted Ultra Detail is printed with wax support for external and internal features, which was removed using a hot water ultrasonic cleaning.

We printed resolution test pieces using the Form1+ and SW-FUD to determine the minimum internal channel dimensions (S2 Fig). The resolution test piece was designed with 10 circular channels ranging in diameter from 1.9 to 0.1 mm and 12 square channels ranging in width from 1.15 to 0.05 mm. All channels were designed to be the length of the test piece, 40 mm. For the Form 1+, the smallest diameter circular channel that cleared was 0.9 mm and the smallest square channel that cleared was 0.65 mm (measurements in CAD). For SW-FUD, the smallest cleared circular channel was 0.3 mm and the smallest cleared square channel was 0.25 mm. These channel dimensions are similar to the minimum channel dimensions reported in previous studies that printed internal channels using SLA and inkjet printing [19,24].

Devices were designed to mix the reagents via co-laminar diffusion and 3D micromixing. The syringe pump pulled reagents into the device from the two inlet chambers. The two reagents in the Form1+ and SW-FUD devices mixed while being pulled through the long channel and during incubation. The design of the Form1+ 3D Micromixer was inspired by previous 3D passive micromixers that have been designed and fabricated using multiple layers of 2D substrates [36].

The Form 1+ devices (Fig 2A and 2B) featured internal channels that were designed to be 1.5 mm in circular diameter and measured to be 1.30 mm (standard deviation: 0.12 mm) (S1 Table). These devices took 3–4 hours to print and the materials cost per device was approximately $1. The SW-FUD Co-Laminar device (Fig 2C) was designed to have a 300-micron square channel and a reactor volume of 910 nL. The average side length of the cross-sections (S3I–S3L Fig) was 220 microns (standard deviation of 60 microns) (S1 Table). Given this average side length, the average reactor volume of the printed SW-FUD devices was 490 nL (standard deviation of 140 nL). The devices cost $5.71 each on Shapeways with a delivery time ranging from 2–10 days.

**Fig 3:**
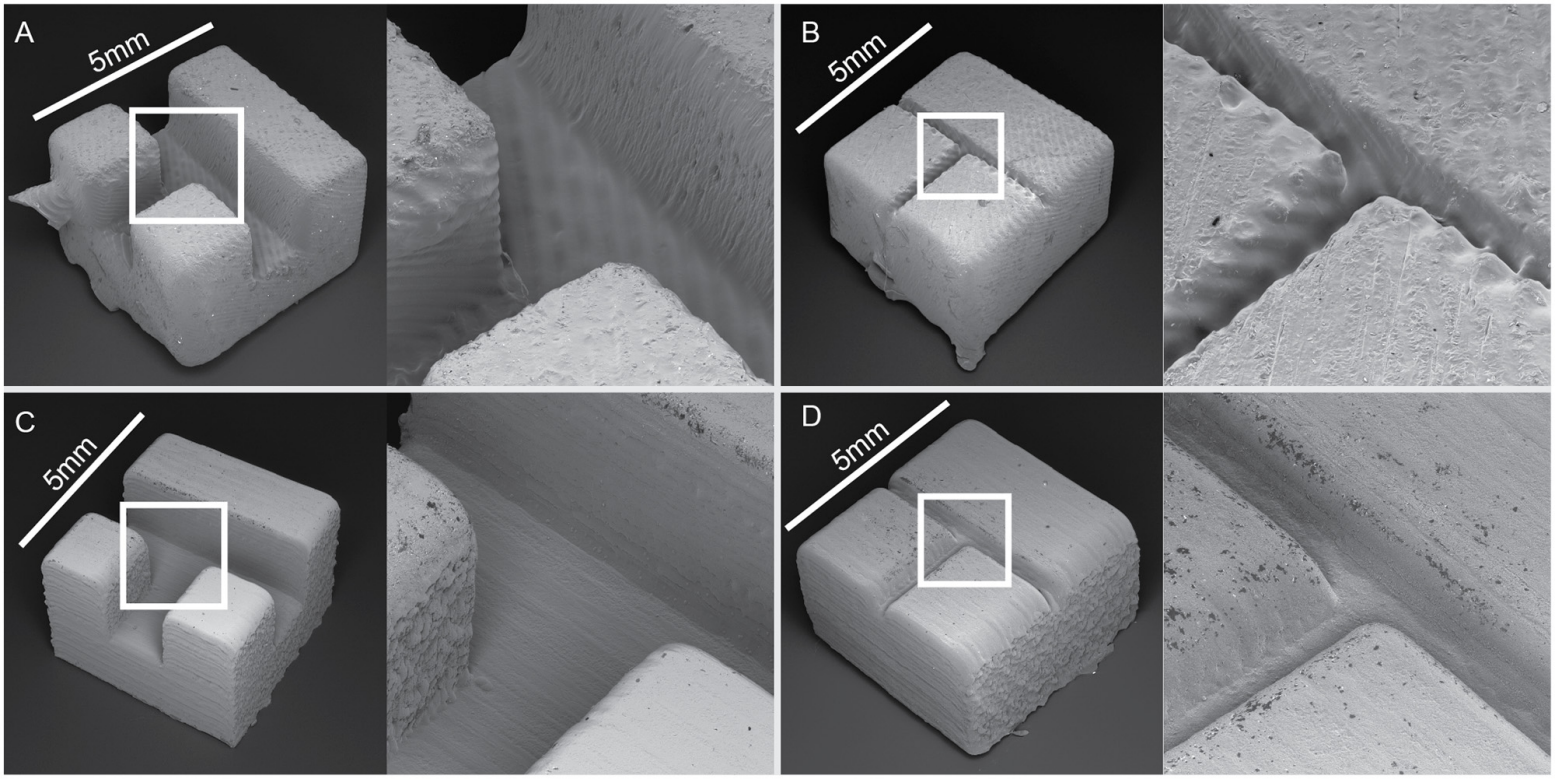
Scanning electron microscopy of open channels printed using the Form1+ (A and B) and Shapeways Frosted Ultra Detail (C and D). Each piece was designed to be 5 mm on a side. Channels were designed to be 1500 microns wide in A & C and 300 microns wide in B & D.

To investigate the surface roughness of each printing method, scanning electron microscope images (SEMs) were taken of two test pieces printed using both fabrication methods. The two test pieces featured open channels designed to be 300 and 1500 microns wide. The open channels printed using SW-FUD (Fig 3C and 3D) were less deformed and showed less surface roughness than parts printed on the Form1+ (Fig 3A and 3B). Striations were clearly visible in all test pieces and their orientation appears to effect surface roughness.

**Fig 4:**
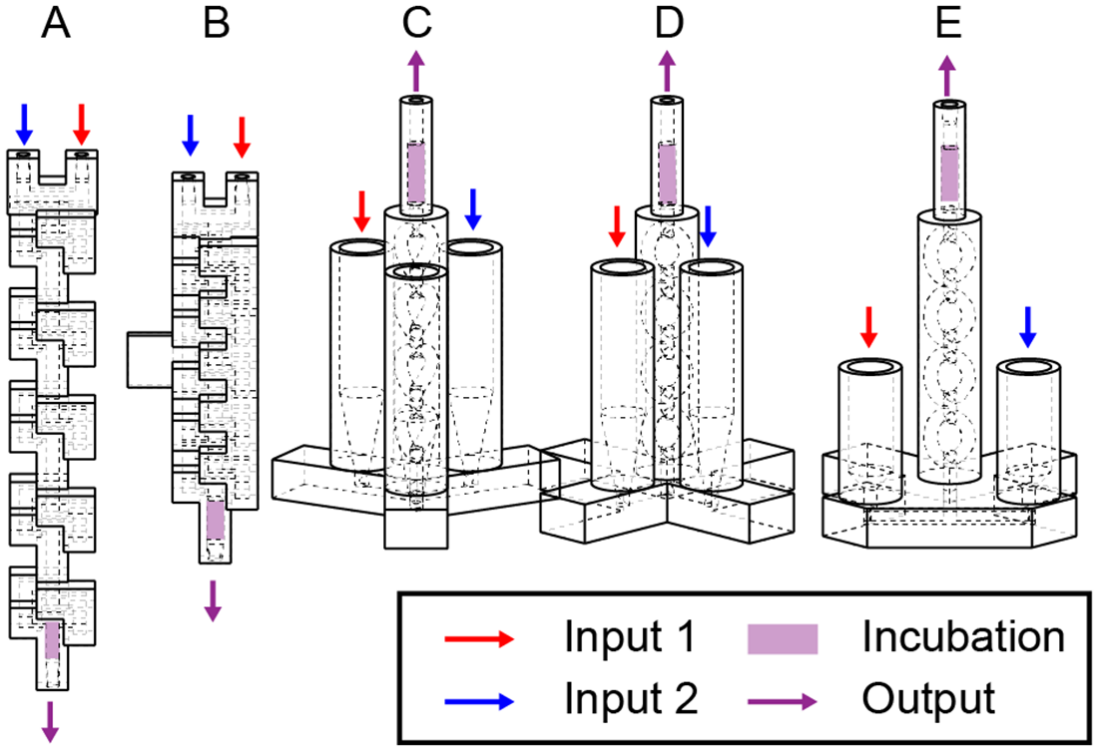
Design evolution of the 3D Micromixer. Many designs were created and tested for each of the three fluidic devices (Fig 2) used in the DNA assembly experiments. Five (A-E) of 13 iterations of the Form 1+ 3D Micromixer (Fig 2B) can be seen above. Several design changes can be seen A-E: The design of the 3D micromixer was changed from a recti-linear channels with square cross-sections (A-B) similar to previous 3d micromixer designs [19,36,37], to a design using a circular cross-section and smooth curves (C-E). A mount held the initial designs (A-B); later versions (C-E) stood freely on a base. All 13 iterations are in S4 Fig

The usability and performance of the fluidic devices were improved through these design iterations. Thirteen versions of the Form 1+ 3D Micromixer, five versions of the Form 1+ Co-Laminar Mixer, and three versions of the SW-FUD Co-Laminar Mixer were designed, fabricated, and tested (S4 Fig). Fig 4 shows five versions of the Form1+ 3D Micromixer. Originally, reagents were pushed through device inlets (Fig 4A and 4B), which required pushing both inputs into the device at the same time. Devices were redesigned to pull reagents from the outlet. To interface tubing (0.060 in OD, Tygon Microbore, Saint-Gobain, Courbevoie, France) with the fluidic hardware, a press-fit interface (1.5 mm hole with 0.15 mm diameter, 98 degree chamfer) was designed. This interface worked reliably for the Form1+ printed parts; however, leaks occasionally occurred in SW-FUD devices, likely due to the higher pressure required to push fluid through the smaller channel. Other studies have 3D-printed standard Luer fittings [15,19,22,23,25] into the devices, which prevent leaks but adds dead volume. A press fit interface for a 23-gauge dispensing needle was also designed in the inlets and outlets of the SW-FUD device. This interface was used to clear wax out of the channels by aspirating the melted wax and hot water through the device.

### Design and testing of the 3D printed syringe pump

We operated the microfluidic devices using an open-source, 3D printed syringe pump (S5–S10 Figs). The syringe pump has three components: the mechanical parts, a circuit board (S9 Fig), and a user interface (S10 Fig). The mechanical parts consisted of a stepper motor, threaded rod, and 3D printed parts. The 3D printed parts were designed in SolidWorks and printed using the Makerbot Replicator 2X. The parts were modular and included parts printed for different syringe sizes (S8 Fig). The material cost of the printed parts was $10.11. The electronics were custom designed in EagleCAD and the circuit board was milled using a Roland Modela MDX-20 desktop CNC mill. Surface mounted components, including an Atmel ATMega328P microcontroller and Allegro A3909 stepper motor driver, were soldered onto the milled circuit board (S9 Fig). Custom Arduino-based firmware was written to take commands from a computer via USB and then control the stepper motor (S6 Fig). A user interface was written in Processing to command the pump (S10 Fig). The user specified the volumetric flow rate and desired flow volume and commanded the syringe pump to either pull or push fluid. The total bill of materials cost of the pump was $56.63 and the full bill of materials is in S2 Table.

Performance and usability improvements were made to the syringe pump through many design iterations. We designed the mechanical components of the syringe pump to assemble without fasteners, making assembly far simpler. This feature along with the custom PCB board lowered costs. The overall bill of materials cost 37–63% less than previously published open-source syringe pumps [32].

### Golden Gate DNA assembly in 3D printed fluidics

For each of the three fluidic designs, we employed Golden Gate DNA assembly to construct a bacterial plasmid from two pieces of linear double stranded DNA. One piece encodes a plasmid backbone with a p15A medium-copy number origin of replication and ampicillin resistance marker and the second piece encodes a constitutive promoter driving the expression of a yellow fluorescent protein (YFP).

Each fluidic device was used to mix two volumes: a solution containing both pieces of DNA in ligase buffer and a solution containing BsaI restriction enzyme and T4 DNA ligase in ligase buffer (Fig 2). The two solutions were drawn into each fluidic device using the 3D printed syringe pump, mixed, and then incubated within the device for 90 minutes at room temperature. We transformed unpurified assembly reactions into *E*. *coli*, plated serial dilutions on agar media with antibiotics for plasmid selection, and then counted the number of colony-forming units (CFUs) for each assembly reaction (Table 1). For all devices, negative controls that included both DNA segments but omitting enzymes yielded no colonies. Positive controls—in which the components were mixed together by pipette and incubated in a PCR tube—yielded an average of 1.60 x 10^5^ CFUs. To investigate whether the 3D printed photopolymer material inhibited the assembly, we incubated reactions that were pre-mixed by pipette in each device. For both the Form 1+ and SW-FUD devices, there was no significant difference in the number of CFUs from these experiments compared to the in-tube positive controls.

**Table 1:** Colony forming units of *E*. *coli* transformed with golden gate products and grown on ampicillin plates for each of the 3D printed fluidics.

Device	Assembly Reaction	No. of Samples	Mean CFU (x 10^5^)	S^a^ (x 10^5^)
Tube control				
	No enzyme	4	0.00	0.00
	Enzyme & DNA	7	1.60	0.80
Form 1+ co-laminar				
	No enzyme	1	0.00	0.00
	Off device mix^b^	6	1.42	0.83
	On device mix^c^	5	1.30	1.20
Form 1+ micromixer				
	No enzyme	1	0.00	0.00
	Off device mix^b^	5	1.74	1.42
	On device mix^c^	7	1.71	1.54
Shapeways co-laminar				
	No enzyme	1	0.00	0.00
	Off device mix^b^	5	1.79	1.67
	On device mix^c^	5	2.53	0.52

^a^. Standard deviation.

^b^. Golden Gate enzymes and DNA mixed off device and incubated on device.

^c^. Golden Gate enzymes and DNA mixed on device and incubated on device.

We counted the number of CFUs yielded from each of the assembly reactions mixed within the microfluidic devices. Colony counts varied significantly from device—to—device, though overall no significant difference was apparent in the number of CFUs compared to the in-tube positive control. The SW-FUD devices that mixed the DNA and enzymes in the device yielded an average of 2.53 x 10^5^ CFUs (Table 1). Due to the high variability in CFUs per transformation for all experiments (Table 1, column S^a^), the data should not be used as a quantitative benchmark comparing the efficacy of DNA assembly between the fluidic devices or between the fluidic devices and the tube control. Rather, we conclude from these experiments that performing Golden Gate assembly in these novel device formats does not seem to significantly interfere with the process compared to standard methods. All data from the experiments are available in the supplementary information (S3 Table). Despite different reactor volumes between the SW-FUD and Form1+ devices, similar ranges of CFUs were observed for each device format, suggesting that the amount of DNA was saturating in the transformation. We grew five fluorescent colonies from Form1+ device assemblies, purified plasmid from the cultures, and sequenced the assembly junctions and YFP insert. The assembly junctions for each plasmid were free of sequence defects.

## Discussion

This work accomplished three goals: (1) demonstrate iterative development of 3D-printed fluidic devices; (2) characterize the performance of two types of 3D printing, the Form1+ SLA Desktop printer and Shapeways Frosted Ultra Detail, for creating micro- and millifluidics; and (3) validate the efficacy of 3D-printed fluidic devices for performing Golden Gate DNA assembly. More broadly, this study shows the compatibility of the devices and materials for enzymatic reactions of interest to synthetic biology.

### Iterative development of 3D printed fluidic devices

Over the course of this project, we demonstrated that the speed, simplicity, and low expense of 3D printing enabled the iterative development of 3D-printed fluidics and syringe pump hardware. Each fluidic design was less than $6 to print and fluidics printed on the Form1+ required a few hours to print. As a result, many iterations of each fluidic device were designed, fabricated and tested. Fig 4 displays some design iterations of the 3D Micromixer, fabricated on the Form 1+ (all design iterations are in S4 Fig). To develop the syringe pump, numerous versions of the mechanical components and PCB board (S9 Fig) were fabricated using 3D printing and CNC milling.

This project is also an example of the development process for open labware [38]. The design, fabrication, and implementation of the 3D-printed fluidic hardware began in an MIT course in January 2014, and then continued as collaboration between three different MIT labs (Lincoln Laboratory, Synthetic Biology Center, Media Lab). These laboratories each had common 3D printing infrastructure, enabling the exchange of digital design files and local printing. Adoption of 3D printers continues to grow, enabling more researchers to design, download, print, and use open labware. The design files for all of the devices presented in this work can be found in ‘Metafluidics’ (www.metafluidics.com), an open repository of design files for microfluidics.

Other than the necessary 3D printing infrastructure, other barriers remain for the co-development of 3D-printed open labware. First, CAD remains a large barrier for many potential designers. Fluidic devices, in particular, are challenging to design because they require complex 3D forms. The authors of this study had multiple years of experience working with SolidWorks. Many biologists interested in designing fluidics will likely not have similar experience. To overcome this barrier, microfluidic-specific 3D CAD tools could be created to make fluidic design more accessible. A tool that uses a library of microfluidic parts that can be dragged and dropped in a visual user interface may be particularly useful. Second, open labware tools that require multiple fabrication machines, such as the syringe pump created for this project, are more challenging to fabricate and require additional skills to build. Creating hardware kits that can be purchased, assembled, and modified may promote the dissemination of these types of tools. The OpenPCR platform [39] is a successful implementation of this strategy.

### Performance of Form1+ and Shapeways-Frosted Ultra Detail for printing micro and millifluidics

The second aim of this work was to characterize the performance of the Form1+ and SW-FUD for printing micro- and millifluidics. Overall, device performance was consistent with previously reported devices printed using stereolithography and inkjet printing. Two reports published during our investigation used digital light projection (DLP) stereolithography to print square fluidic channels as small as 200–300 microns wide [15,19], smaller than the minimum square channel dimension, 650 microns, printed using the Form 1+. Instead of DLP, the Form 1+ uses galvanometers to steer laser light into the unpolymerized resin. This suggests that desktop DLP stereolithography machines, such as the MiiCraft [40] (MiiCraft, Taiwan), Titan 1 [41] (Kudo3D, Pleasanton, CA), and ProJet 1200 [42] (3D Systems, Rock Hill, South Carolina), may be more effective than the Form1+ for printing the smallest internal channel dimensions. However, the Form1+ has a larger build area than these desktop DLP printers and has begun marketing flexible resins, which could be used to print elastomeric fluidic devices. Although Shapeways does not publicly state the machines they use for their various materials, the SW-FUD material is likely printed using the 3D Systems ProJet HD 3000 (3D Systems, Rock Hill, South Carolina)[43], a high-resolution inkjet printer. A previous report used this printer to make internal square channels between 100–1000 microns [24], consistent with our results.

For both the SW-FUD and Form1+ devices, the geometry of the internal channel cross sections varied from part to part (S3 Fig). This finding was similar to reports from previous studies [15,19]. One study found that print orientation affects the geometry of the printed channels [15]. Print orientation was not controlled in the fabrication of the Form1+ devices and was unknown for the SW-FUD devices. Therefore, it is possible that print orientation was a contributing factor for part-to-part variability.

### Efficacy of Form1+ and SW-FUD fluidic devices for performing Golden Gate DNA assembly

The assembly products from both the Form 1+ and SW-FUD fluidic devices yielded tens of thousands of transformants. Both pre-mix and on-device mix conditions produced similar or greater numbers of transformants than in-tube controls. These results demonstrate a proof-of-principle that 3D printed devices can adequately mix the Golden Gate enzymes and DNA and that the Golden Gate biochemistry proceeds successfully in these materials with these geometries. However, we did not find differences between the fluidic devices and the positive control in assembly efficacy. Future work could investigate differences in assembly efficacy across the design parameter space—e.g. material choice, fabrication method, internal diameter and geometry—by using quantitative PCR of the assembled junction versus the backbone.

In this work, we assembled two pieces of DNA in the 3D-printed fluidic devices—a method that could easily be done in a larger test tube by hand. However, the devices could be extended in future work to improve the utility of this method versus a manual workflow. First, the Golden Gate biochemistry is extensible to larger assemblies and has been used to generate constructs with many pieces [7,9,10]. Thus, these devices or similar ones could be used to assemble more complex genetic circuits using the same method. Secondly, these devices could be integrated into a full synthetic biology workflow that includes PCR of linear segments of DNA and bacterial transformation. Separate 3D-printed parts could be designed for each of these steps and then integrated into a full workflow. Similar integrated, 3D-printed fluidic systems have recently been reported but have not been used for biochemical reactions with DNA [18,24]. Programmable, multi-step fluid operations could be handled using 3D printed valves, which have also recently been demonstrated [20,21,44].

In addition to printing fluidics used in a synthetic biology workflow, 3D printing could provide an alternative method for manufacturing disposable microfluidic chips used in point-of-care diagnostics [45,46], including nucleic acid analysis [47–49]. Many disposable microfluidic devices are manufactured using injection molding and hot embossing, enabling low per-unit cost at high volumes. However, initial tooling costs are high, around $15,000 for a single layer device [15], increasing costs for low to medium volume production. 3D printing is an attractive, alternative manufacturing approach for low-to-medium volumes given the low initial cost of desktop 3D printers, low per-unit device cost, and the ability to rapidly iterate on device design.

## Conclusions

This project demonstrated that the Shapeways-Frosted Ultra Detail and Form1+ 3D-printed devices are compatible with Golden Gate DNA assembly reactions. This finding is a step towards creating open, inexpensive and rapidly produced 3D-printed microfluidic devices that can perform a synthetic biology workflow. The project also demonstrated how 3D printing enables open, collaborative and rapid design of fluidics. Limitations currently exist with printing fluidics using desktop 3D printers and on-demand printing services, particularly print resolution and part-to-part variability. However, 3D printing technology is rapidly improving due to considerable academic and commercial interest and these limitations could be overcome as the technology develops.

## Materials and Methods

### Designing & 3D printing fluidic devices

Fluidic devices were designed using SolidWorks (Dassault Systèmes SolidWorks Corp, Waltham, Massachusetts). Micro and millifluidic CAD designs were printed using two approaches: (1) desktop stereolithography printing using the Form1+ (Formlabs, Somerville, Massachusetts) and (2) on-demand printing using Shapeways (Shapeways, New York, New York) Frosted Ultra Detail (SW-FUD) material.

STL files were generated in Solidworks. For 3D printing on the Form 1+, STL files were opened in Form Lab’s PreForm software. The software was used to automatically orient the devices and generate supports. All devices printed on the Form1+ were printed using Form Labs Clear Resin (formulation: FLGPCL02) with a z-resolution of 0.05 mm. For 3D printing using Shapeways, STLs were uploaded to www.shapeways.com and Frosted Ultra Detail material was selected. For printing, the “print it anyway” option was selected. All design files can be found in ‘Metafluidics’ (www.metafluidics.com), an open-repository of design files for fluidic systems.

### Post-processing devices to clear channels

To process parts printed on the Form1+ (Fig 3A and 3B), devices were removed from the build tray and submerged in 91% isopropyl alcohol for 3–5 minutes. Compressed air was blown into each inlet for 10 seconds to remove alcohol and uncured resin. Devices were then submerged in 91% isopropyl alcohol again for 1 minute. A syringe was used to wash each channel with isopropyl alcohol. Then, compressed air was blown into each inlet for 10 seconds. Finally, support material on each device was removed.

SW-FUD devices (Fig 3C) were submerged in hot water in a Magnasonic ultrasonic cleaner to clear wax support material. The devices were cleaned for several minutes. While in the ultrasonic cleaner, a 1 cc syringe (BD, Franklin Lakes, New Jersey) with a 23 gauge, ½” dispensing needle was used to dispense hot water through the channels. After cleaning, channels were tested by hand using the same syringe and needle.

### Measuring device cross sections

Cross sections were prepared by cutting post-processed devices using high leverage handheld metal snips. The sections were sanded using a belt sander. Liquid was dispensed and compressed air was blown through the sectioned channel to remove any debris created from sanding.

To determine the dimensions of the internal channels of the Form 1+ and SW-FUD devices, 4 cross section samples were measured for each device type using a USB optical microscope (SuperEyes B008, Shenzhen D&F, Shenzhen, China). Images were measured in ImageJ. To determine the scale of each image, a known length on the image was measured using digital calipers.

For 8 Form1+ samples, each circular cross section was measured 3 times. For 4 SW-FUD samples, the square side length was measured 6 times, 3 times horizontally and 3 vertically. To estimate the experimental error for each sample, we calculated the standard deviation of the technical replicates. The reaction volume of each SW-FUD sample was calculated by squaring the side length and multiplying by the expected length of the channel.

### Examining surface roughness with electron microscopy

To examine the surface roughness of the two printing methods, two small test pieces were designed. The test pieces featured open channels of 300 and 1500 microns. For electron microscopy, representative samples of each printing method for each test piece were sputter coated with gold and examined with a Tescan Vega GMU Scanning Electron Microscope (SEM).

### Design, fabrication, testing & operation of the syringe pump

The syringe pump was designed in SolidWorks. A MakerBot Replicator 2 (Makerbot Industries, Brooklyn, New York) was used to print parts in polylactic acid (PLA). The pump used a NEMA 17 bipolar stepping motor with 200 steps per revolution (Evil Mad Scientist LLC, Sunnyvale, California), a 3/8” threaded rod with 1/12” in pitch (McMaster-Carr, Elmhurst, Illinois) and a custom electronics board designed in Eagle (Cadsoft Computer GmbH, Pleiskirchen, Germany). To create the circuit board, 4” x 6” FR1 circuit board blanks (Inventables, Chicago, Illionis) were milled using a Roland Modela MDX-20 CNC mill (Roland DG Corporation, Hamamatsu, Japan). The electronic components were then soldered into place. The circuit board was connected to a laptop computer by USB FTDI cable. The Atmel Atmega328P microcontroller was bootloaded with Arduino firmware using a AVR in-system programmer. A full bill of materials of the pump is in S3 Table. The circuit board was then programmed and powered via USB. A user interface for the pump was written in Processing. To operate the syringe pump, the user inputted their desired flow rate and volume into the interface and then pressed “pull” or “push” to move the pump. Operation of the pump is diagrammed in S6 Fig.

### Preparing fluidic devices for biological protocol

Devices printed on the Form1+ were additionally cured under a 15 watt fluorescent UV lamp for 24 hrs. 30 minutes prior to running the biological experiment, both devices were blocked with bovine serum albumin (BSA). BSA was added by hand into each device using a syringe. After 30 minutes, BSA was removed from the channels by using pressurized air.

### World-to-device interfacing

Inlets of all fluidic devices were designed to allow pipetting reagents into inlet wells. Outlets were designed to press-fit with a Tygon Microbore tube (0.060” outer diameter). The press-fit outlet-tube connection was checked for leaks. In the case of a leaky fit, parafilm was used to create an airtight fit. The outlet tube (Tygon Microbore, 0.060” OD, 10–15 cm long) was connected to a 23-gauge, ½” dispensing needle tip & syringe (1 mL BD Luer Lok) and loaded into the syringe pump.

### Amplification and purification of DNA parts

The plasmid backbone encoding an ampicillin-resistance cassette [50] and a p15A origin of replication [51] was PCR amplified using primers that comprised (from 5’ to 3’): a BsaI restriction enzyme recognition site, a 4bp assembly scar, and a backbone-annealing region. Forward primer: CGCGGGGGTCTCCAATGCCGTCTTCGCTTCCTCGCTC. Reverse primer: GGTGCAGGTCTCGAAGCTGGTCTTCCAGTACAATCTGCTCTGATG.

The YFP cassette encoding the bacterial P_Tac_ promoter [52], ribozyme insulator RiboJ [52], ribosomal binding site B0064 [53], yellow fluorescent protein coding sequence [54], and transcriptional terminator L3S2P21 [55] was amplified using primers that comprised a BsaI restriction enzyme recognition site, a 4bp assembly scar, and an insert-annealing region. Forward primer: CTAGCGGGTCTCAGCTTAACGATCGTTGGCTGTGTTGACAATTAATCATCGGC; reverse primer: TGCCCAGGTCTCTCATTGGACCAAAACGAAAAAAGGCCC.

PCR mixtures were set up that contained 25 μL KAPA HiFi HotStart ReadyMix (Kapa Biosystems, Wilmington, MA), 100 pmol of each DNA oligonucleotide primer (Integrated DNA Technologies, Coralville, IA), 23 μL of sterile water, and 0.1 ng of double-stranded DNA template. The reactions were thermocylced according to the following program: (1) 95°C for 3 min, (2) 98°C for 20 seconds, (3) 65°C for 15 seconds, (4) 72°C for 1 minute, (5) repeat steps 2–5 25 times, (6) 72°C for 2 min. PCR amplicons were electrophoresed on a 1% agarose gel, excised with a scalpel, and purified using a Zymoclean Gel DNA Recovery Kit (Zymo Research, Irvine, CA) and eluted in sterile water. The concentration of each purified PCR product was measured using a NanoDrop 1000 Spetrophotometer (NanoDrop, Wilmington, DE).

### Golden Gate assembly setup

The Golden Gate assembly reaction was set up into two reaction halves: the first containing DNA in ligase buffer, and the second containing enzymes in ligase buffer. These reaction halves were mixed in subsequent steps. Enzymes and buffer were purchased from New England Biolabs, Ipswich, MA.

DNA solution composition (per reaction):

40 fmol linear plasmid backbone amplicon
40 fmol linear YFP insert
1 μL 10X T4 DNA ligase reaction buffer
Sterile H_2_O to 10 μL

Enzyme solution composition (per reaction):

1 μL BsaI
1 μL T4 DNA ligase
1 μL 10X T4 DNA ligase reaction buffer
7 μL sterile H_2_O

### Experimental protocol for devices printed using the Form 1+

Before beginning experiments, devices were printed, processed, prepared for the biological protocol and interfaced with tubes to the syringe pump (see protocols above). 10 μL of DNA solution and 10 μL of enzyme solution were pipetted into separate inlets. 40 μL of mineral oil was pipetted on top of each solution. The reagents were then drawn through the device using the syringe pump at a rate of 5 μL per second and allowed to incubate at the end of the device near the outlet. After incubation, the reaction product was drawn into the outlet tube with the syringe by hand and immediately pushed into a collection tube and heat inactivated at 80°C. For each device, we also tested pre-mixed reactions. 10 μL of DNA solution and 10 μL of enzyme solution were mixed using a pipette in a 1.5 mL Eppendorf tube. The pre-mix was then pipetted into one inlet. 80 μL of oil was pipetted on top of the mixed reagents. The other inlet was capped using parafilm. Pre-mixed reactions were similarly drawn through the device and into the incubation area and allowed to incubate for 90 minutes. A negative control was completed using the same protocol by substituting the 10 μL of enzyme solution with 10 μL of 1X ligase buffer.

### Experimental protocol for devices printed using Shapeways Frosted Ultra Detail

SW-FUD devices were cleared, prepared for biological protocol and interfaced with tubes to the syringe pump. 5 μL of DNA solution and 5 μL of enzyme solution were pipetted into separate inlets. 10 μL of mineral oil was pipetted on top of both solutions to minimize evaporative losses. Using the syringe pump, the two solutions were drawn through the device at a rate of 1 μL per second until liquid emerged from the outlet tube. After a 90-minute incubation, the outlet tube was removed and replaced with a new tube. Then, the reaction product was drawn out of the device by hand using a syringe and immediately pushed into a collection tube and heat-inactivated at 80°C.

Pre-mix reactions and negative controls were also completed in the SW-FUD devices. For the pre-mix reactions, 5 μL of DNA solution and 5 μL of enzyme solution were mixed by micropipette and then pipetted into one inlet. The other inlet was covered. 10 μL of mineral oil was pipetted on top. The mixture was drawn into the device at 1 μL per second until it could be seen in the outlet tube. Post-incubation, the outlet tube was replaced and the remaining pre-mix reaction and oil were removed from the inlet. 10 μL of de-ionized water was added to the inlet. The reaction product in the device and the DI water in the inlet were then drawn into the outlet, pushed into a collection tube and heat-inactivated at 80 C. A negative control was completed using the same protocol, except 5 μL of ligase buffer was substituted for the enzyme solution. Detailed visual protocols for SW-FUD devices can be found in S1 Fig.

### Transformation and counting colony-forming units

Heat-inactivated assembly mixtures were transformed into One Shot Mach1 T1 Phage-Resistant Competent Cells (*E*. *coli* genotype: F- φ80(lacZ)ΔM15 ΔlacX74 hsdR(rK-mK+) ΔrecA1398 endA1 tonA; Life Technologies, Carlsbad, CA). 0.6–5.0 μL of assembly mixture was added to 50 μL of thawed cells, incubated on ice for 35 minutes, incubated at 42°C for 30 seconds, placed back on ice for 2 minutes, and then 450 μL of SOC media were added to the cells. The cells were allowed to recover for 1 hour in a shaking incubator at 37°C. 50 μL of recovery outgrowth was diluted ten-fold into 450 μL SOC three times to obtain 1X, 10X, 100X and 1000X dilutions. 150 μL of each dilution was plated onto an LB agar plate with 100 μg/mL ampicillin and allowed to grow overnight at 37°C.

The following day, the number of colonies that grew on each agar plate corresponding to the 1000X dilution was tallied.

For sequencing, fluorescent colonies were picked and plasmid DNA was purified. The following primers were used for sequencing: Forward: CGGTCACAGCTTGTCTGTAAGC. Reverse: CATAGTAAGCCAGTATACACTCCGCTAG

## Supporting Information

S1 Fig Visual protocol for running pre-mix and on device mix for the SW-FUD device. (PDF)

S2 Fig Form 1+ and SW-FUD resolution test piece. (A) CAD file of resolution test piece. Side views of printed Form 1+ (B) and SW-FUD (C) resolution test pieces. Visualization of cleared circular fluid channels in the Form 1+ (D) and SW-FUD (E) resolution test pieces. Close-in images of a resolution test piece printed on the Form 1+ (F-J) and SW-FUD (K-O). Scale bar: 1 mm. All images except D & E were taken using a SuperEyes B008 USB Microscope (Shenzhen D&F Co, Ltd, Shenzhen, China). D & E were taken using a Canon EOS 7D Digital SLR camera.
(PDF)

S3 Fig Cross sections of fluidic devices. Cross-sections were taken of the Form1+ Co-Laminar Mixer devices (A-D), Form 1+ 3D Micromixer devices (E-H), and SW-FUD Co-Laminar Mixer device (I-L). Scale bar: 0.5 mm. All images were taken using a SuperEyes B008 USB Microscope (Shenzhen D&F Co, Ltd, Shenzhen, China). Measurements of the channel dimension (diameter for the circle & side length for the square channel) can be found in Table S1.
(PDF)

S4 Fig Design iterations of each fluidic device. SW-FUD Co-Laminar Mixer (Top), Form 1+ Co-Laminar Mixer (Middle), and Form 1+ 3D Micromixer (Bottom). The rightmost iteration of each design was used for DNA assembly.
(PDF)

S5 Fig 3D printed syringe pump. A bespoke syringe pump and user interface was designed and fabricated for this experiment. The syringe pump was used to pull the two inputs through the device at 1 and 5 uL/sec. The syringe pump is composed of several parts (S7 Fig), including 3D printed mechanical pieces, a custom designed and fabricated circuit board (S9 Fig), and a software user interface (S10 Fig). The CAD designs and software for the syringe pump have been made openly available. The total cost of the pump was $56.33 (S2 Table) and the 3D printed components can each be printed in less than 3 hours. Photo credit: Taylor Levy and Che-Wei Wang.
(PDF)

S6 Fig Operation of the syringe pump. From Top to Bottom: A user interface (UI) (S10 Fig) was designed in Processing and enabled the user to control the syringe pump. Depending on the desired pumping direction, flow rate & volume and the hardware settings (syringe ID, thread dimension, steps per revolution of the motor), the UI sent a G CODE command to the electronics firmware with the desired motor rotation direction, speed and number of steps. The circuit board (S9 Fig) received the G Code command and translated it into electronic motor controls that moved the stepper a certain number of steps at the desired speed and in the desired direction. Each motor step moved the syringe a small distance; pushing a small amount of fluid in or out of the dispensing needle top.
(PDF)

S7 Fig Syringe pump components. From top to bottom: USB-FTDI cable; 3D printed circuit board case top and bottom (optional); Milled electronic control board; Bi-polar stepper motor, threaded rod & 3D printed adapter; 3D printed base; 3D printed mid-section; 3D printed top; 2-1cc syringes with 23 gauge luer lock ½” dispensing needles connected to 3D printed fluidic device using 0.060” OD Tygon Microbore tubing.
(PDF)

S8 Fig Modular components of the syringe pump. (Left) The base of the pump includes the Nema 17 stepper motor, a 3D printed connector, and the 3D printed base. The motor press fits into the 3D printed base. The 1cc configuration was used in the DNA assembly experiments. The Mid & Top pieces were modified to accommodate 10 and 100cc syringes.
(PDF)

S9 Fig Syringe pump control board. The circuit board traces were milled using a Roland Modela MDX-20 CNC mill. Serial commands from the Processing UI and 5V DC were transmitted to the circuit board via FTDI USB TTL Serial. The board used a ATMega 328P (Atmel, San Jose, California) microcontroller that was programmed via an AVRISP. The ATMega 328P was bootloaded with Arduino and used the AccelStepper library to send commands to an Allegro A3909 Dual Stepping Motor driver. A heat sink was attached to the top of the A3909 driver. The arduino code is available in the online supplementary information.
(PDF)

S10 Fig Syringe pump user interface. The interface was written using Processing and communicated to the circuit board (S9 Fig) via USB TTL Serial commands. In the interface, the user specified parameters of the syringe pump: thread size, steps per revolution of the motor, and the inner diameter of the syringe. These values were used to compute a volume moved per step. To control the pump, the user inputted a desired flow rate & flow volume and then clicked “Pull” or “Push” to move the pump accordingly. The processing code is available in the online supplementary information.
(PDF)

S1 File Syringe pump software (SI_Arduino_Code.ino, SI_Processing_Code.pde), STL files for the syringe pump (SI_Base.STL, SI_Case_Bottom, SI_Case_Top.STL, SI_FL_CL.STL, SI_Mid-1cc.STL, SI_Top-1cc.STL), STL file for Shapeways Frosted Ultra Detail co-laminar mixer (SI_SW-FUD_CL.STL), STL files for Formlabs co-laminar and micromixer (SI_FL_CL.STL, SI_FL_MM.STL), Resolution test piece for printing characterization (SI_ResolutionTest.STL), and printing test pieces for SEM imaging (SI_300_test.STL & SI_1500_test.STL).
(ZIP)

S1 Table Dimensions of SW-FUD and Form 1+ cross sections (S3 Fig). (PDF)

S2 Table Syringe pump bill of materials. This does not take into account the upfront cost of buying machines and tools used to fabricate the pump. The cost of the 3D printed syringe pump components was calculated by measuring the mass of filament used and multiplying that by the material price per unit mass.
(PDF)

S3 Table Full dataset. (PDF)
