# Supplementary figures and images for: DNA Assembly in 3D Printed Fluidics
Source: PLoS One. 2015 Dec 30;10(12):e0143636. doi: 10.1371/journal.pone.0143636 (PMC4699221; doi:10.1371/journal.pone.0143636)

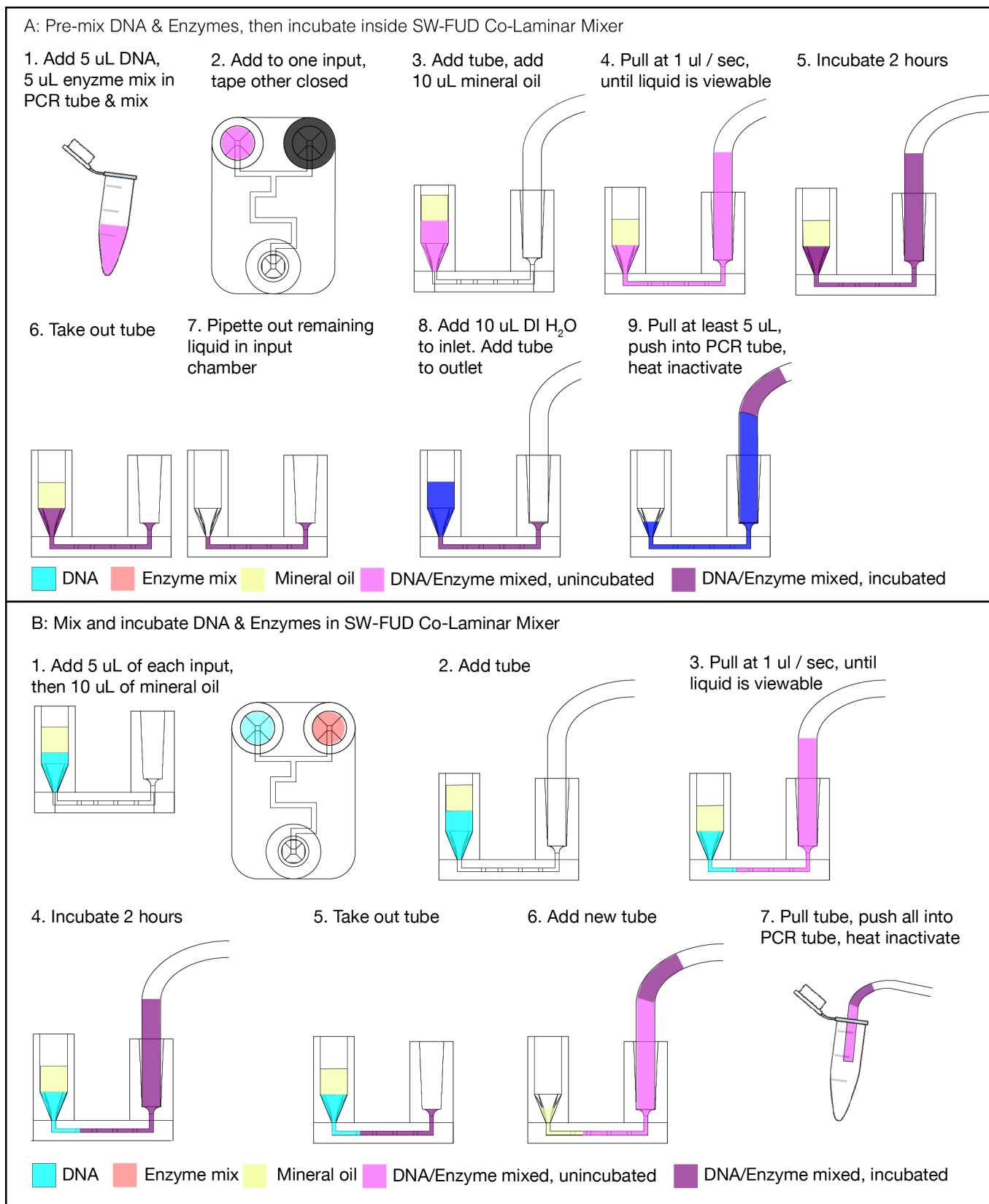

**Fig. S1** | Visual protocol for running pre-mix and on device mix for the SW-FUD device.

Supplement: S1 Fig — (PDF) [file pone.0143636.s001.pdf]
