# Supplementary material for: DNA Assembly in 3D Printed Fluidics
Source: PLoS One. 2015 Dec 30;10(12):e0143636. doi: 10.1371/journal.pone.0143636 (PMC4699221; doi:10.1371/journal.pone.0143636)
Supplement: S2 Fig — (A) CAD file of resolution test piece. Side views of printed Form 1+ (B) and SW-FUD (C) resolution test pieces. Visualization of cleared circular fluid channels in the Form 1+ (D) and SW-FUD (E) resolution test pieces. Close-in images of a resolution test piece printed on the Form 1+ (F-J) and SW-FUD (K-O). Scale bar: 1 mm. All images except D & E were taken using a SuperEyes B008 USB Microscope (Shenzhen D&F Co, Ltd, Shenzhen, China). D & E were taken using a Canon EOS 7D Digital SLR camera. (PDF) [file pone.0143636.s002.pdf]

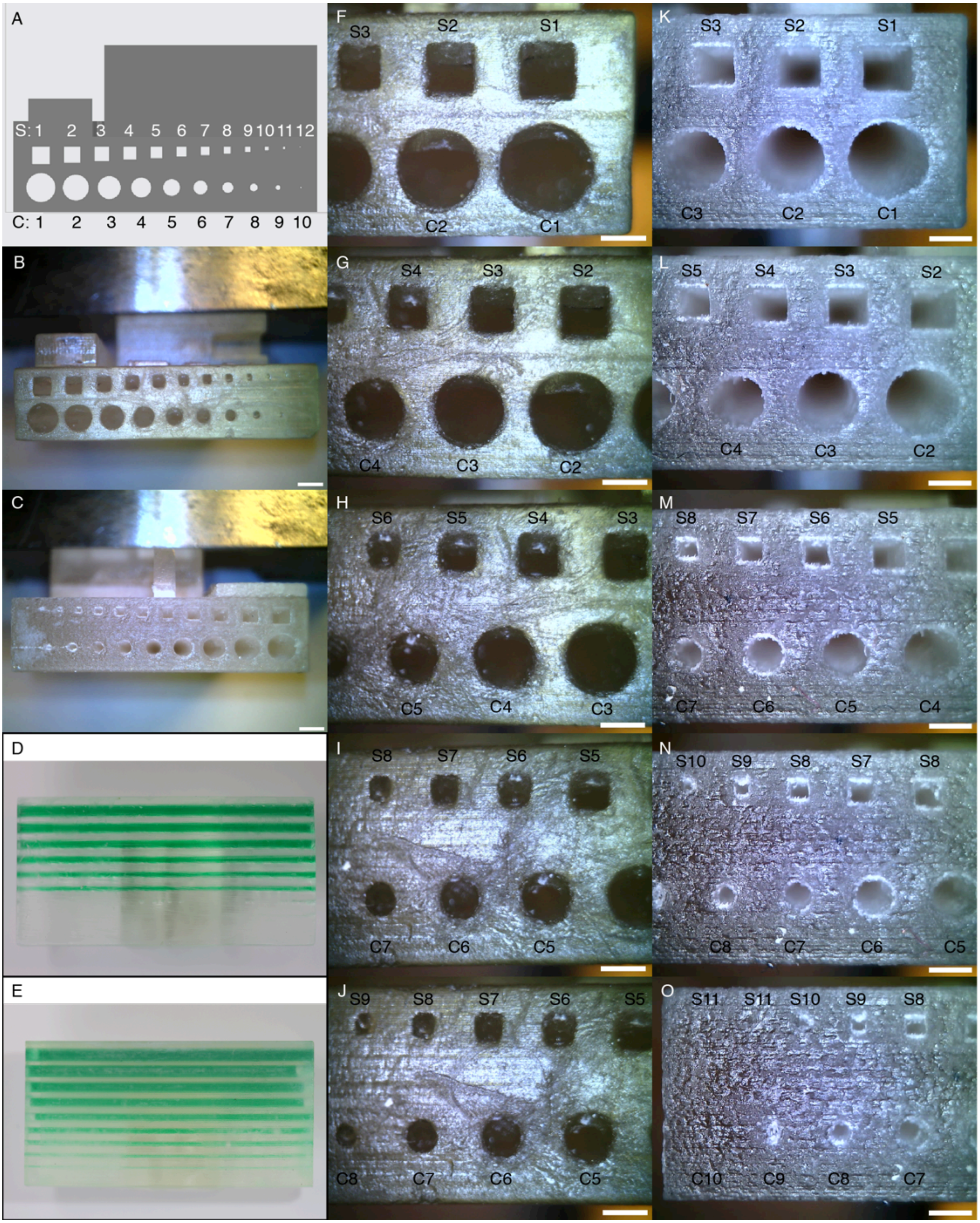

Fig. S2 | Form 1+ and SW-FUD resolution test piece. (**A**) CAD file of resolution test piece. Side views of printed Form 1+ (**B**) and SW-FUD (**C**) resolution test pieces. Visualization of cleared circular fluid channels in the Form 1+ (**D**) and SW-FUD (**E**) resolution test pieces. Close-in images of a resolution test piece printed on

the Form 1+ (**F-J**) and SW-FUD (**K-O**). Scale bar: 1 mm. All images except **D & E** were taken using a SuperEyes B008 USB Microscope (Shenzhen D&F Co, Ltd, Shenzhen, China). **D & E** were taken using a Canon EOS 7D Digital SLR camera.
