# Supplementary material for: DNA Assembly in 3D Printed Fluidics
Source: PLoS One. 2015 Dec 30;10(12):e0143636. doi: 10.1371/journal.pone.0143636 (PMC4699221; doi:10.1371/journal.pone.0143636)
Supplement: S3 Fig — Cross-sections were taken of the Form1+ Co-Laminar Mixer devices (A-D), Form 1+ 3D Micromixer devices (E-H), and SW-FUD Co-Laminar Mixer device (I-L). Scale bar: 0.5 mm. All images were taken using a SuperEyes B008 USB Microscope (Shenzhen D&F Co, Ltd, Shenzhen, China). Measurements of the channel dimension (diameter for the circle & side length for the square channel) can be found in Table S1. (PDF) [file pone.0143636.s003.pdf]

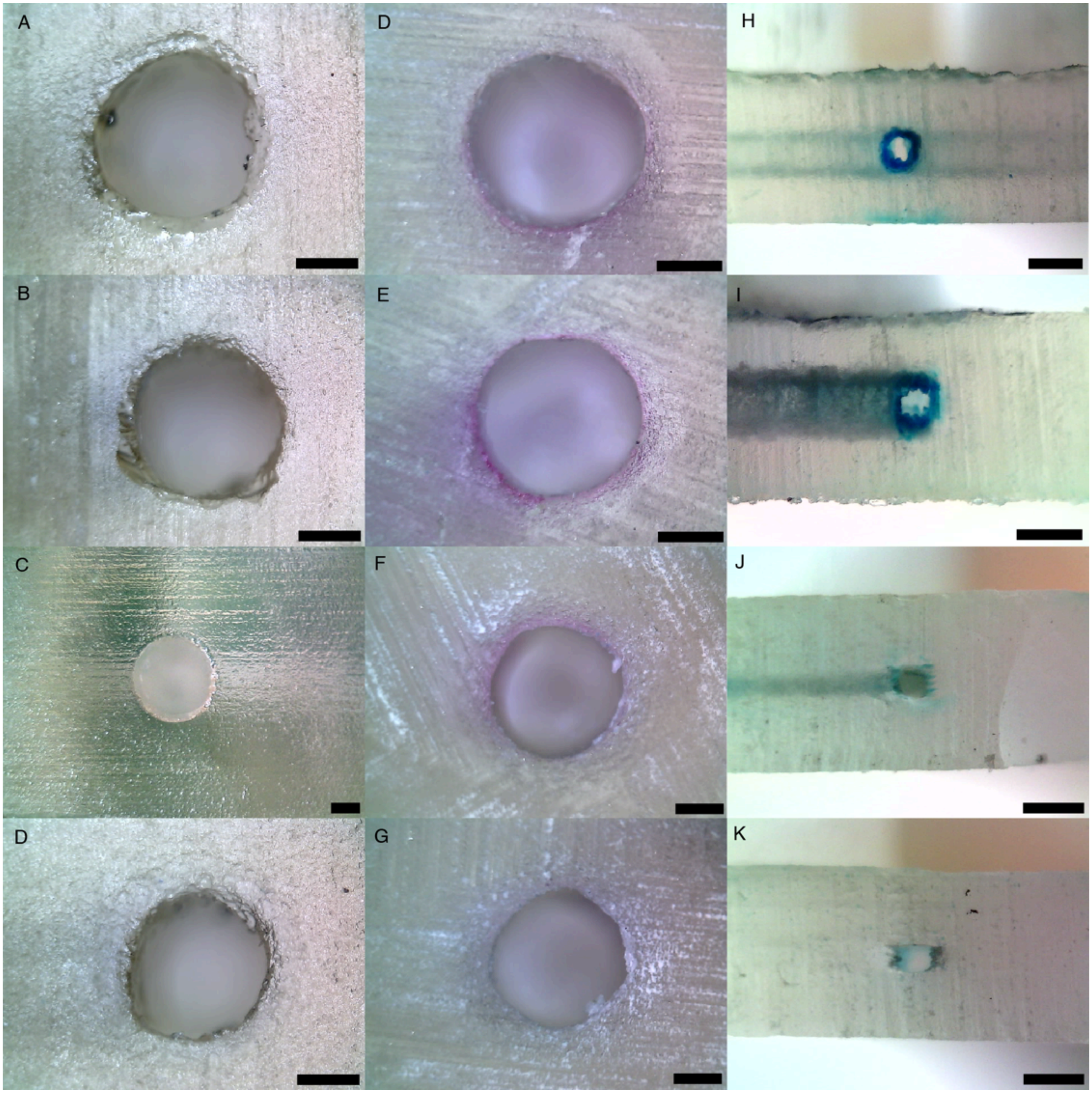

Fig. S3 | **Cross sections of fluidic devices.** Cross-sections were taken of the Form1+ Co-Laminar Mixer devices (**A-D**), Form 1+ 3D Micromixer devices (**E-H**), and SW-FUD Co-Laminar Mixer device (**I-L**). Scale bar: 0.5 mm. All images were taken using a SuperEyes B008 USB Microscope (Shenzhen D&F Co, Ltd, Shenzhen, China). Measurements of the channel dimension (diameter for the circle & side length for the square channel) can be found in **Table S1.**
