# Supplementary material for: DNA Assembly in 3D Printed Fluidics
Source: PLoS One. 2015 Dec 30;10(12):e0143636. doi: 10.1371/journal.pone.0143636 (PMC4699221; doi:10.1371/journal.pone.0143636)
Supplement: S4 Fig — SW-FUD Co-Laminar Mixer (Top), Form 1+ Co-Laminar Mixer (Middle), and Form 1+ 3D Micromixer (Bottom). The rightmost iteration of each design was used for DNA assembly. (PDF) [file pone.0143636.s004.pdf]

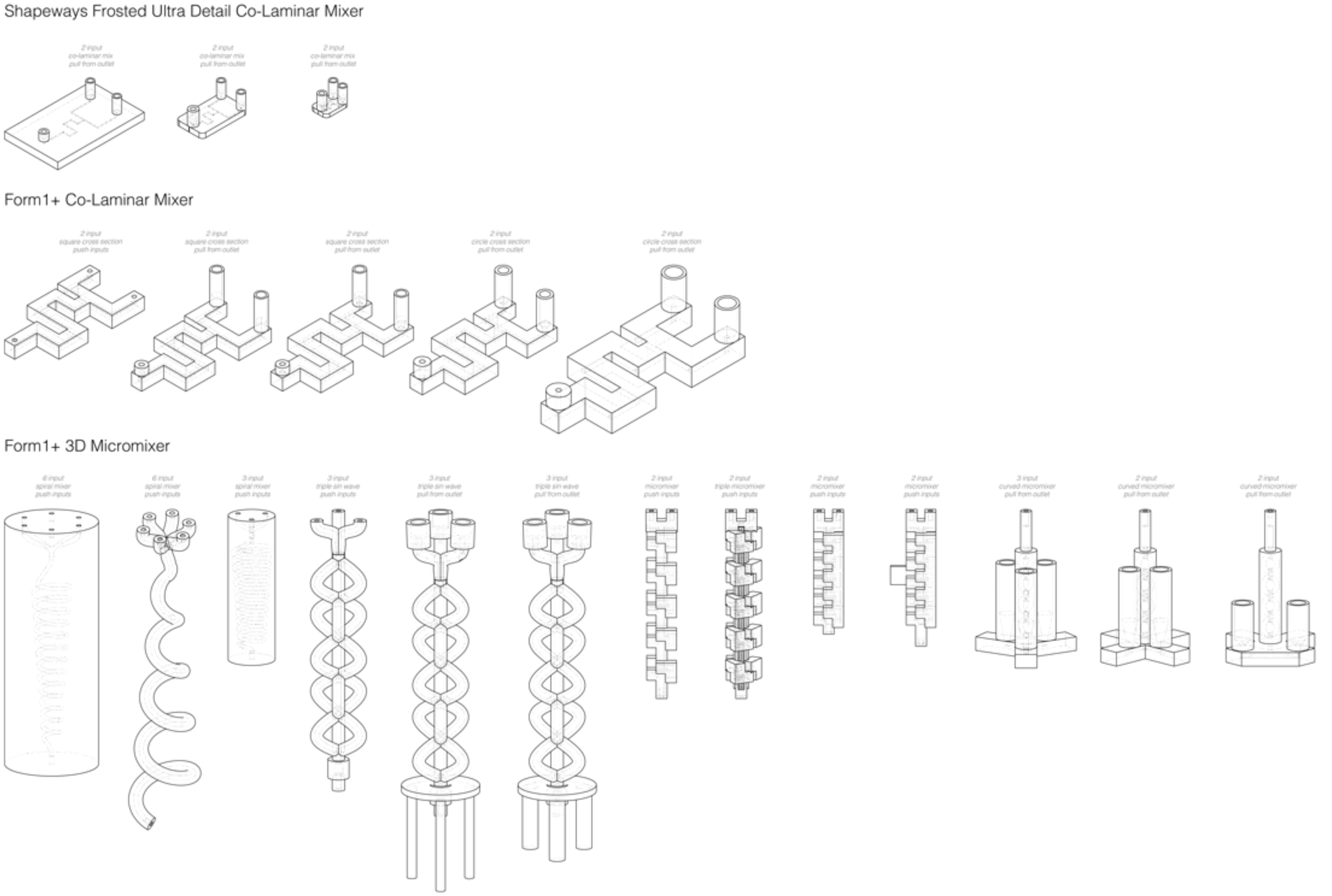

Fig. S4 | **Design iterations of each fluidic device**. SW-FUD Co-Laminar Mixer (Top), Form 1+ Co-Laminar Mixer (Middle), and Form 1+ 3D Micromixer (Bottom). The rightmost iteration of each design was used for DNA assembly.
