# Supplementary material for: DNA Assembly in 3D Printed Fluidics
Source: PLoS One. 2015 Dec 30;10(12):e0143636. doi: 10.1371/journal.pone.0143636 (PMC4699221; doi:10.1371/journal.pone.0143636)
Supplement: S5 Fig — A bespoke syringe pump and user interface was designed and fabricated for this experiment. The syringe pump was used to pull the two inputs through the device at 1 and 5 uL/sec. The syringe pump is composed of several parts (S7 Fig), including 3D printed mechanical pieces, a custom designed and fabricated circuit board (S9 Fig), and a software user interface (S10 Fig). The CAD designs and software for the syringe pump have been made openly available. The total cost of the pump was $56.33 (S2 Table) and the 3D printed components can each be printed in less than 3 hours. Photo credit: Taylor Levy and Che-Wei Wang. (PDF) [file pone.0143636.s005.pdf]

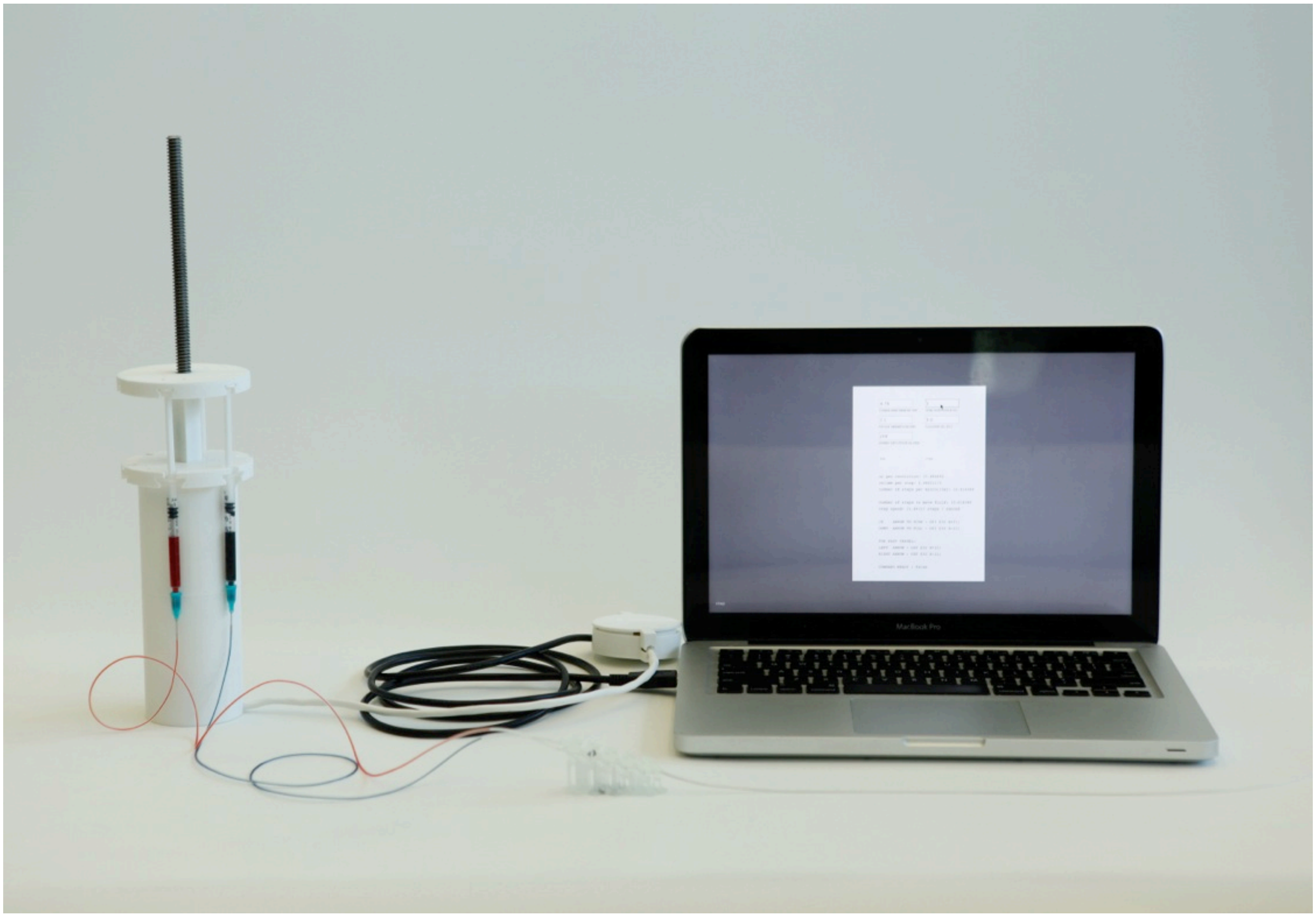

Fig. S5 | 3D printed syringe pump. A bespoke syringe pump and user interface was designed and fabricated for this experiment. The syringe pump was used to pull the two inputs through the device at 1 and 5 uL/sec. The syringe pump is composed of several parts (**Fig. S7**), including 3D printed mechanical pieces, a custom designed and fabricated circuit board (**Fig. S9**), and a software user interface (**Fig. S10**). The CAD designs and software for the syringe pump have been made openly available. The total cost of the pump was $56.33 (**Table S2**) and the 3D printed components can each be printed in less than 3 hours. Photo credit: Taylor Levy and Che-Wei Wang.
