# Supplementary material for: DNA Assembly in 3D Printed Fluidics
Source: PLoS One. 2015 Dec 30;10(12):e0143636. doi: 10.1371/journal.pone.0143636 (PMC4699221; doi:10.1371/journal.pone.0143636)
Supplement: S6 Fig — From Top to Bottom: A user interface (UI) (S10 Fig) was designed in Processing and enabled the user to control the syringe pump. Depending on the desired pumping direction, flow rate & volume and the hardware settings (syringe ID, thread dimension, steps per revolution of the motor), the UI sent a G CODE command to the electronics firmware with the desired motor rotation direction, speed and number of steps. The circuit board (S9 Fig) received the G Code command and translated it into electronic motor controls that moved the stepper a certain number of steps at the desired speed and in the desired direction. Each motor step moved the syringe a small distance; pushing a small amount of fluid in or out of the dispensing needle top. (PDF) [file pone.0143636.s006.pdf]

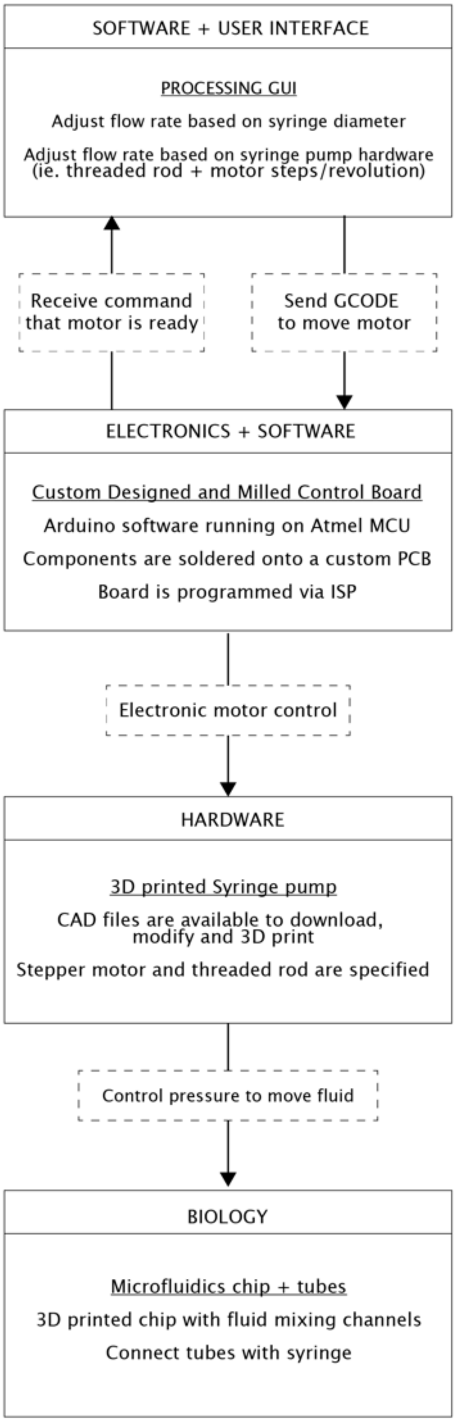

Fig. S6 | **Operation of the syringe pump.** From Top to Bottom: A user interface (UI) (**Fig. S10**) was designed in Processing and enabled the user to control the syringe pump. Depending on the desired pumping direction, flow rate & volume and the hardware settings (syringe ID, thread dimension, steps per revolution of the motor), the UI sent a G CODE command to the electronics firmware with the desired motor rotation direction, speed and number of steps. The circuit board (**Fig. S9**) received the G Code command and translated it into electronic motor controls that moved the stepper a certain number of steps at the desired speed and in the desired direction. Each motor step moved the syringe a small distance; pushing a small amount of fluid in or out of the dispensing needle top.
