# Supplementary material for: DNA Assembly in 3D Printed Fluidics
Source: PLoS One. 2015 Dec 30;10(12):e0143636. doi: 10.1371/journal.pone.0143636 (PMC4699221; doi:10.1371/journal.pone.0143636)
Supplement: S7 Fig — From top to bottom: USB-FTDI cable; 3D printed circuit board case top and bottom (optional); Milled electronic control board; Bi-polar stepper motor, threaded rod & 3D printed adapter; 3D printed base; 3D printed mid-section; 3D printed top; 2-1cc syringes with 23 gauge luer lock ½” dispensing needles connected to 3D printed fluidic device using 0.060” OD Tygon Microbore tubing. (PDF) [file pone.0143636.s007.pdf]

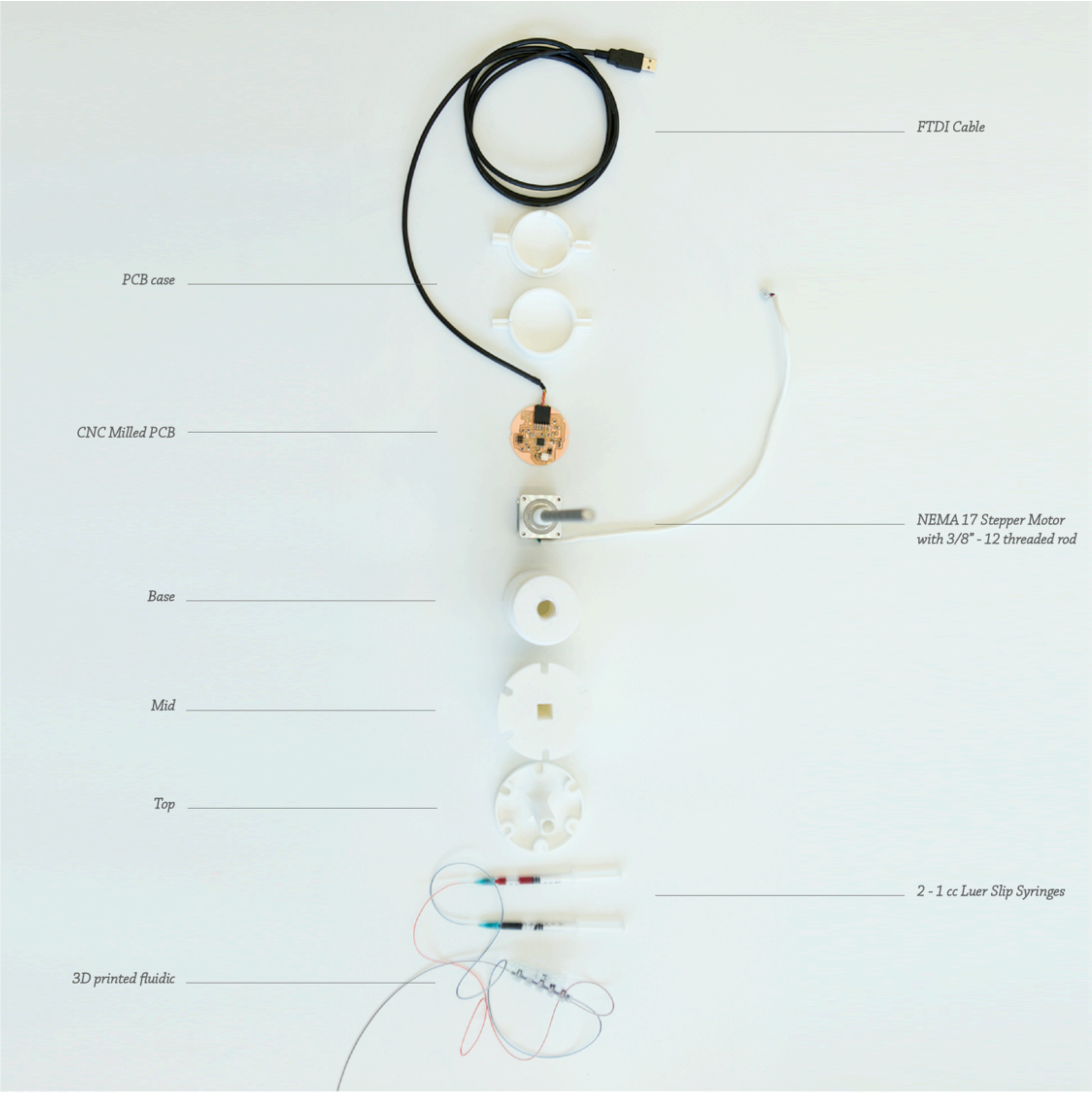

Fig. S7 | Syringe pump components. From top to bottom: USB-FTDI cable; 3D printed circuit board case top and bottom (optional); Milled electronic control board; Bi-polar stepper motor, threaded rod & 3D printed adapter; 3D printed base; 3D printed mid-section; 3D printed top; 2-1cc syringes with 23 gauge luer lock ½" dispensing needles connected to 3D printed fluidic device using 0.060" OD Tygon Microbore tubing.
