# Supplementary material for: DNA Assembly in 3D Printed Fluidics
Source: PLoS One. 2015 Dec 30;10(12):e0143636. doi: 10.1371/journal.pone.0143636 (PMC4699221; doi:10.1371/journal.pone.0143636)
Supplement: S8 Fig — (Left) The base of the pump includes the Nema 17 stepper motor, a 3D printed connector, and the 3D printed base. The motor press fits into the 3D printed base. The 1cc configuration was used in the DNA assembly experiments. The Mid & Top pieces were modified to accommodate 10 and 100cc syringes. (PDF) [file pone.0143636.s008.pdf]

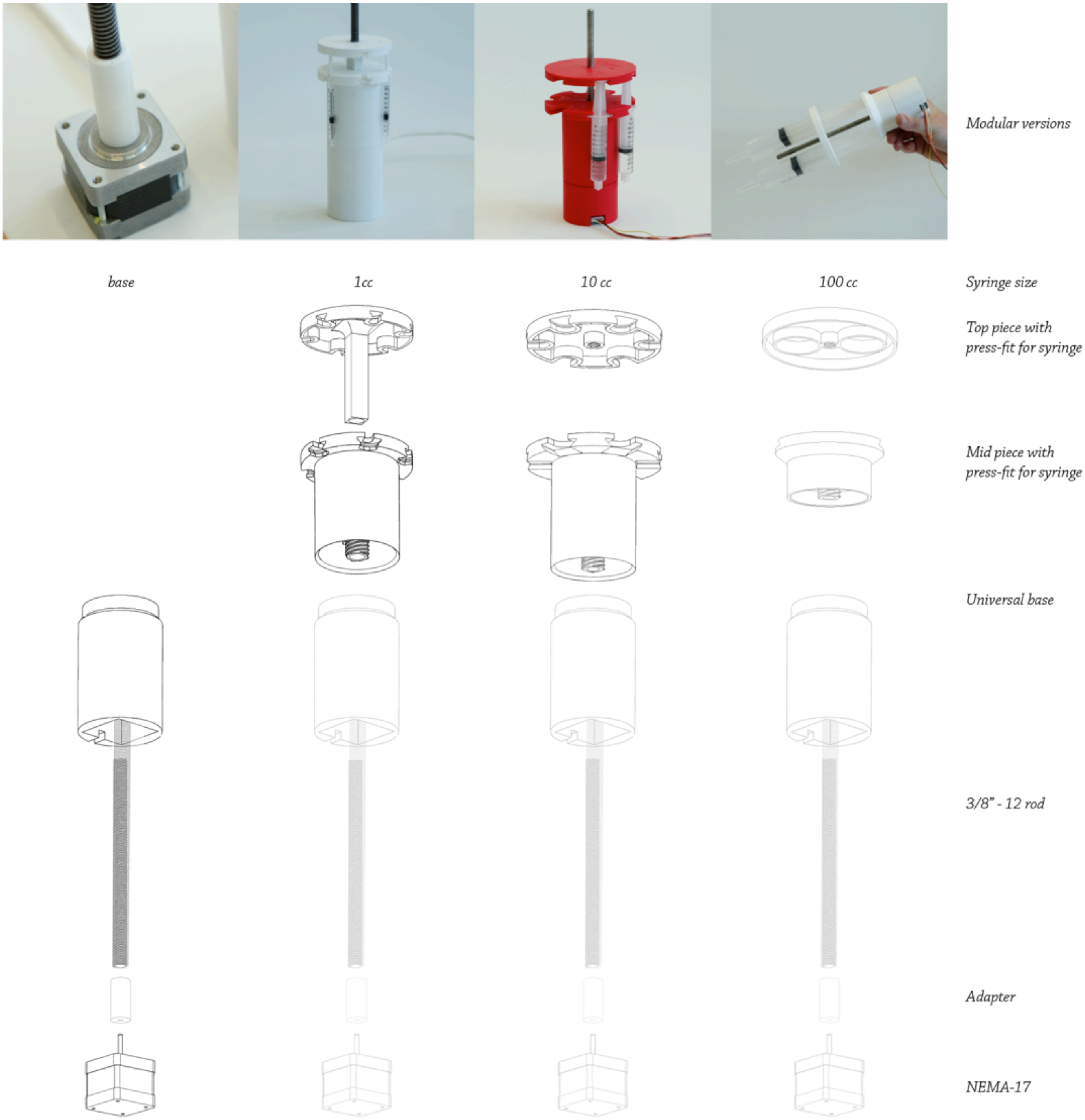

Fig. S8 | **Modular components of the syringe pump**. (**Left**) The base of the pump includes the Nema 17 stepper motor, a 3D printed connector, and the 3D printed base. The motor press fits into the 3D printed base. The 1cc configuration was used in the DNA assembly experiments. The Mid & Top pieces were modified to accommodate 10 and 100cc syringes.
