# Supplementary material for: DNA Assembly in 3D Printed Fluidics
Source: PLoS One. 2015 Dec 30;10(12):e0143636. doi: 10.1371/journal.pone.0143636 (PMC4699221; doi:10.1371/journal.pone.0143636)
Supplement: S9 Fig — The circuit board traces were milled using a Roland Modela MDX-20 CNC mill. Serial commands from the Processing UI and 5V DC were transmitted to the circuit board via FTDI USB TTL Serial. The board used a ATMega 328P (Atmel, San Jose, California) microcontroller that was programmed via an AVRISP. The ATMega 328P was bootloaded with Arduino and used the AccelStepper library to send commands to an Allegro A3909 Dual Stepping Motor driver. A heat sink was attached to the top of the A3909 driver. The arduino code is available in the online supplementary information. (PDF) [file pone.0143636.s009.pdf]

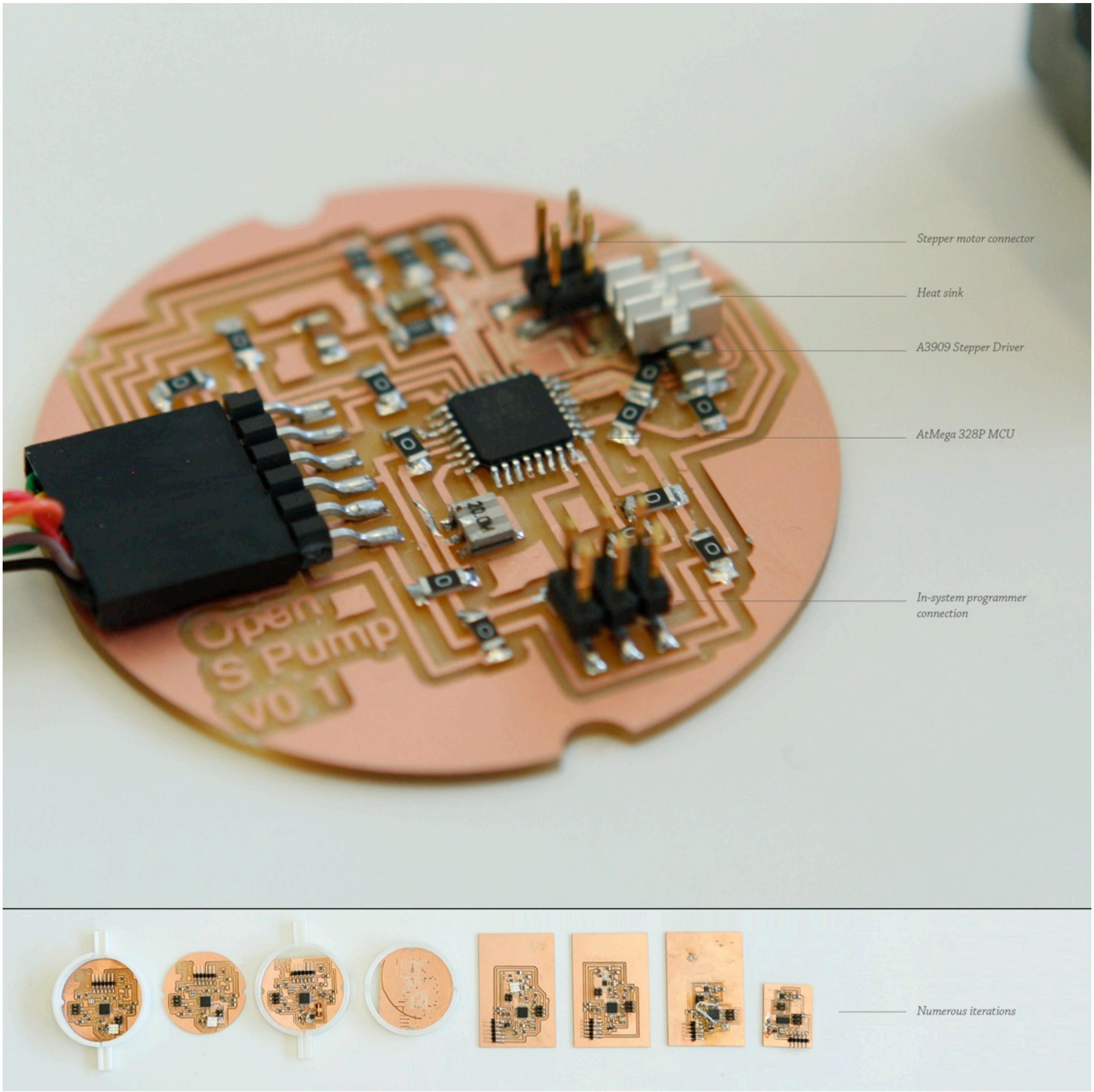

Fig. S9 | **Syringe pump control board**. The circuit board traces were milled using a Roland Modela MDX-20 CNC mill. Serial commands from the Processing UI and 5V DC were transmitted to the circuit board via FTDI USB TTL Serial. The board used a ATMega 328P (Atmel, San Jose, California) microcontroller that was programmed via an AVRISP. The ATMega 328P was bootloaded with Arduino and used the AccelStepper library to send commands to an Allegro A3909 Dual Stepping Motor driver. A heat sink was attached to the top of the A3909 driver. The arduino code is available in the online supplementary information.
