# Supplementary material for: DNA Assembly in 3D Printed Fluidics
Source: PLoS One. 2015 Dec 30;10(12):e0143636. doi: 10.1371/journal.pone.0143636 (PMC4699221; doi:10.1371/journal.pone.0143636)
Supplement: S10 Fig — The interface was written using Processing and communicated to the circuit board (S9 Fig) via USB TTL Serial commands. In the interface, the user specified parameters of the syringe pump: thread size, steps per revolution of the motor, and the inner diameter of the syringe. These values were used to compute a volume moved per step. To control the pump, the user inputted a desired flow rate & flow volume and then clicked “Pull” or “Push” to move the pump accordingly. The processing code is available in the online supplementary information. (PDF) [file pone.0143636.s010.pdf]

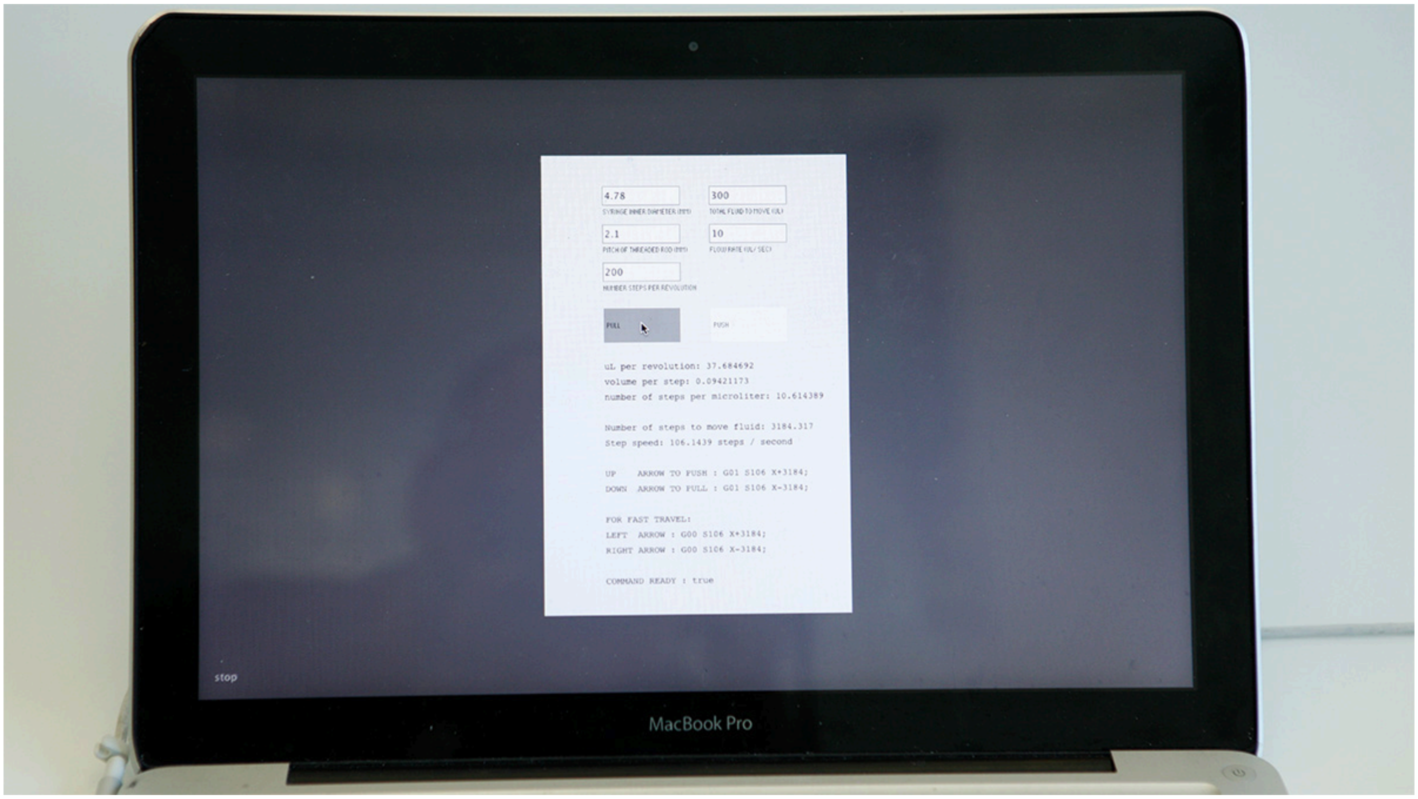

Fig. S10 | **Syringe pump user interface**. The interface was written using Processing and communicated to the circuit board (**Fig. S9**) via USB TTL Serial commands. In the interface, the user specified parameters of the syringe pump: thread size, steps per revolution of the motor, and the inner diameter of the syringe. These values were used to compute a volume moved per step. To control the pump, the user inputted a desired flow rate & flow volume and then clicked “Pull” or “Push” to move the pump accordingly. The processing code is available in the online supplementary information.
