# Supplementary material for: DNA Assembly in 3D Printed Fluidics
Source: PLoS One. 2015 Dec 30;10(12):e0143636. doi: 10.1371/journal.pone.0143636 (PMC4699221; doi:10.1371/journal.pone.0143636)
Supplement: S1 Table — (PDF) [file pone.0143636.s012.pdf]

Table S1 | Dimensions of SW-FUD and Form 1+ cross sections (Fig. S3).

Device type	D (mm)
**Form1+**[a]	
Expected	1.5
A	1.31 ± 0.01[c]
B	1.26 ± 0.04[c]
C	1.40 ± 0.02[c]
D	1.14 ± 0.02[c]
E	1.37 ± 0.01[c]
F	1.28 ± 0.02[c]
G	1.36 ± 0.04[c]
H	1.40 ± 0.06[c]
Average	1.30
Standard deviation	0.12[d]
**SW-FUD**[b]	
Expected	0.3
I	0.19 ± 0.01[c]
J	0.20 ± 0.01[c]
K	0.23 ± 0.03[c]
L	0.26 ± 0.04[c]
Average	0.22
Standard deviation	0.06[d]

a: The Form1+ devices had circular channels. 3 technical replicates were collected for each circular cross section

b: SW-FUD devices had square channels. 6 techincal replicates were collected for each cross-section, 3 vertically & 3 horizontally.

c. Experimental error estimate calculated using the technical replicates

d. The standard deviation of the sample population, incorporating the experimental error estimate for each sample.
