# Supplementary material for: DNA Assembly in 3D Printed Fluidics
Source: PLoS One. 2015 Dec 30;10(12):e0143636. doi: 10.1371/journal.pone.0143636 (PMC4699221; doi:10.1371/journal.pone.0143636)
Supplement: S2 Table — This does not take into account the upfront cost of buying machines and tools used to fabricate the pump. The cost of the 3D printed syringe pump components was calculated by measuring the mass of filament used and multiplying that by the material price per unit mass. (PDF) [file pone.0143636.s013.pdf]

Table S2 | Syringe pump bill of materials. This does not take into account the upfront cost of buying machines and tools used to fabricate the pump. The cost of the 3D printed syringe pump components was calculated by measuring the mass of filament used and multiplying that by the material price per unit mass.

3D Printed Parts	
Syringe Pump Mid-section	$3.19
Syringe Pump Top	$1.19
Syringe Pump Base	$5.06
Syringe Pump threaded rod adapter	$0.67
	$10.11
**Mechanical parts**	
1cc BD Luer Lock Syringe - 2	$0.77
23 gauge 1/2" dispensing needle - 1	$0.18
Tygon Microbore Tubing, 0.060" OD - 6"	$0.41
Bi-polar Nema 17 stepper motor	$14.00
6" Threaded rod - 3/8" - 12	$3.34
	$18.70
**Electronics**	
PCB board	$1.00
A3909 Dual Stepper Driver	$1.66
ATMega328P	$3.61
FTDI cable	$17.95
0 Ohm resistor x 15	$0.07
10K SMD resistor	$0.01
1 uF ceramic SMD capacitor	$0.07
0.1 uF ceramic SMD capacitor	$0.12
10 uF ceramic SMD capacitor	$0.19
20 Mhz resonator	$0.43
FTDI header	$0.55
2x2 Bergstik pin header	$0.65
2x3 pin header	$0.72
Aluminum heat sink	$0.03
2x2 IDC ribbon cable connector	$0.48
	$27.52
Total:	$56.33
