# Supplementary material for: DNA Assembly in 3D Printed Fluidics
Source: PLoS One. 2015 Dec 30;10(12):e0143636. doi: 10.1371/journal.pone.0143636 (PMC4699221; doi:10.1371/journal.pone.0143636)
Supplement: S3 Table — (PDF) [file pone.0143636.s014.pdf]

Table S3 | Full Dataset

**Device**	**In-tube reaction**			
**Assembly Reaction**	**Enzyme & DNA**			
Assembly reaction volume (uL)	Assembly product transformed (uL)	Colonies (1000x dilution)	Estimated Total CFUs***	CFU/uL assembly product transformed
10	5	13	43333	8667
10	5	36	120000	24000
10	5	42	140000	28000
10	5	61	203333	40667
10	5	76	253333	50667
10	5	77	256666	51333
10	5	31	103333	20667
		MEAN	1.60E+05	3.20E+04
		STD	8.04E+04	1.61E+04
**Device**	**Form 1+ co-laminar**			
**Assembly Reaction**	**Off-device mix**			
Assembly reaction volume (uL)	Assembly product transformed (uL)	Colonies (1000x dilution)	Estimated Total CFUs***	CFU/uL assembly product transformed
10	5	33	110000	22000
10	5	53	176666	35333
10	5	84	280000	56000
10	5	37	123333	24667
10	5	40	133333	26667
10	3.2	9	30000	9375
		MEAN	1.42E+05	2.90E+04
		STD	8.27E+04	1.57E+04
**Device**	**Form 1+ co-laminar**			
**Assembly Reaction**	**On-device mix**			
Assembly reaction volume (uL)	Assembly product transformed (uL)	Colonies (1000x dilution)	Estimated Total CFUs***	CFU/uL assembly product transformed
10	5	46	153333	30667
10	5	23	76667	15333
10	5	99	330000	66000
10	5	12	40000	8000

10	5	15	50000	10000
		MEAN	1.30E+05	2.60E+04
		STD	1.20E+05	2.41E+04
**Device**	**Form 1+ micromixer**			
**Assembly Reaction**	**Off-device mix**			
Assembly reaction volume (uL)	Assembly product transformed (uL)	Colonies (1000x dilution)	Estimated Total CFUs***	CFU/uL assembly product transformed
10	5	70	233100	46620
10	5	30	99900	19980
10	5	119	396270	79254
10	5	19	63270	12654
10	5	23	76590	15318
		MEAN	1.74E+05	3.48E+04
		STD	1.42E+05	2.83E+04
**Device**	**Form 1+ micromixer**			
**Assembly Reaction**	**On-device mix**			
Assembly reaction volume (uL)	Assembly product transformed (uL)	Colonies (1000x dilution)	Estimated Total CFUs***	CFU/uL assembly product transformed
10	5	79	263070	52614
10	5	43	143190	28638
10	5	144	479520	95904
10	5	24	79920	15984
10	5	9	29970	5994
10	5	31	103230	20646
10	5	29	96570	19314
		MEAN	1.71E+05	3.42E+04
		STD	1.54E+05	3.08E+04
**Device**	**Shapeways co-laminar mixer**			
**Assembly Reaction**	**Off-device mix**			
Assembly reaction volume (uL)*	Assembly product transformed (uL) **	Colonies (1000x dilution)	Estimated Total CFUs***	CFU/uL assembly product transformed
0.94	0.336	72	239976	714822
0.94	0.443	37	123321	278128

0.94	0.797	132	439956	552285
0.94	0.553	16	53328	96444
0.94	0.627	11	36630	58452
		MEAN	1.79E+05	3.40E+05
		STD	1.67E+05	2.86E+05
**Device**	**Shapeways co-laminar mixer**			
**Assembly Reaction**	**On-device mix**			
Assembly reaction volume (uL)*	Assembly product transformed (uL) **	Colonies (1000x dilution)	Estimated Total CFUs***	CFU/uL assembly product transformed
0.49	0.490	57	189981	387716
0.49	0.415	99	329967	794614
0.49	0.288	80	266640	925078
0.49	0.258	68	226644	878824
0.49	0.314	76	253080	805724
		MEAN	2.53E+05	7.58E+05
		STD	5.19E+04	2.14E+05
*For the Shapeways co-laminar mixer, assembly reaction volumes correspond to the volume inside the device. See S1 for a visual explanation				
** For the Shapeways co-laminar mixer, DI water was flushed into the reactor to collect the assembly product. The "assembly product transformed" was calculated by dividing the assembly reaction volume by the volume collected out of the device (assembly reaction + DI water) and multiplying by the volume that was then transformed (5 uL).				
*** Estimated total CFU for entire reaction volume				
